# Population cost-effectiveness of the Triple P parenting programme for the treatment of conduct disorder: an economic modelling study

**DOI:** 10.1007/s00787-017-1100-1

**Published:** 2017-12-29

**Authors:** Filipa Sampaio, Jan J. Barendregt, Inna Feldman, Yong Yi Lee, Michael G. Sawyer, Mark R. Dadds, James G. Scott, Cathrine Mihalopoulos

**Affiliations:** 1 Department of Public Health and Caring Sciences (IFV), Uppsala University, BMC, Husargatan 3, 751 22 Uppsala, Sweden; 2 Epigear International, Sunrise Beach, QLD Australia; 3 School of Public Health, The University of Queensland, Herston, QLD Australia; 4 Queensland Centre for Mental Health Research (QCMHR), The Park Centre for Mental Health, Wacol, QLD Australia; 5 School of Medicine, University of Adelaide, Adelaide, SA Australia; 6 Research and Evaluation Unit, Women’s and Children’s Health Network, Adelaide, SA Australia; 7 Child Behaviour Research Clinic, University of Sydney, Sydney, Australia; 8 The University of Queensland Centre for Clinical Research, Herston, QLD Australia; 9 Metro North Mental Health, Royal Brisbane and Women’s Hospital, Herston, QLD Australia; 10 School of Health and Social Development, Deakin Health Economics, Deakin University, Melbourne, Australia

**Keywords:** Population model, Conduct disorder, Children and adolescents, Cost-effectiveness, Parenting programme

## Abstract

Parenting programmes are the recommended treatments of conduct disorders (CD) in children, but little is known about their longer term cost-effectiveness. This study aimed to evaluate the population cost-effectiveness of one of the most researched evidence-based parenting programmes, the Triple P—Positive Parenting Programme, delivered in a group and individual format, for the treatment of CD in children. A population-based multiple cohort decision analytic model was developed to estimate the cost per disability-adjusted life year (DALY) averted of Triple P compared with a ‘no intervention’ scenario, using a health sector perspective. The model targeted a cohort of 5–9-year-old children with CD in Australia currently seeking treatment, and followed them until they reached adulthood (i.e., 18 years). Multivariate probabilistic and univariate sensitivity analyses were conducted to incorporate uncertainty in the model parameters. Triple P was cost-effective compared to no intervention at a threshold of AU$50,000 per DALY averted when delivered in a group format [incremental cost-effectiveness ratio (ICER) = $1013 per DALY averted; 95% uncertainty interval (UI) 471–1956] and in an individual format (ICER = $20,498 per DALY averted; 95% UI 11,146–39,470). Evidence-based parenting programmes, such as the Triple P, for the treatment of CD among children appear to represent good value for money, when delivered in a group or an individual face-to-face format, with the group format being the most cost-effective option. The current model can be used for economic evaluations of other interventions targeting CD and in other settings.

**Electronic supplementary material:**

The online version of this article (10.1007/s00787-017-1100-1) contains supplementary material, which is available to authorized users.

## Background

Conduct disorder (CD) is common in children and adolescents, with a global prevalence of 3.6% of boys and 1.5% of girls aged 5–19 [1]. CD is characterized by, ‘a repetitive and persistent pattern of behaviour in which the basic rights of others or major age-appropriate societal norms or rules are violated’ [2, 3], and is associated with poor educational outcomes, antisocial and criminal behaviour, substance abuse, poor mental health [4, 5], and unemployment [6, 7]. These outcomes place a high burden on individuals, families, and society [8]. Romeo et al. estimated the mean annual cost of a child aged 3–8 with severe antisocial behaviour in the United Kingdom (UK) was of £5960 (in 2002 prices), in terms of health care, educational, and voluntary sector service use, with the greatest cost burden being borne by the family [9]. In a UK longitudinal study of children aged 10 with CD, the costs at age 28 were 10 times higher than those of children with no conduct problems in terms of health service use, educational, and justice system costs [10].

Parenting programmes targeted at parents of children with conduct problems are the recommended treatments of CD [11, 12]. These aim to improve parenting styles and parent–child relationships through the reduction of harsh and inconsistent parenting practices and promote the use of positive incentives and enhanced parent–child communication [13]. One of the most widely researched parenting programmes is the Triple P—Positive Parenting Programme [14]. Triple P is a behavioural family intervention, which aims to prevent and/or treat severe behavioural, emotional, and developmental problems in children and adolescents by enhancing the knowledge, skills, and confidence of parents. It has five levels of increasing intensity, to address the range of symptom severity of behavioural problems. This intervention comprises a variety of strategies targeting both low- and high-risk families, as well as children with problems in the clinical range, and is one of the most comprehensive and systematized parenting programmes available. There is evidence supporting the effectiveness [15–17] and cost-effectiveness [12, 18] of Triple P on child behaviour problems over the short term, but little is known about its long-term cost-effectiveness. Bonin et al., modelled the costs and longer term savings of parenting programmes that could be achieved by reducing the probability of persistent CD among children in the UK, and found them to be cost saving [19]. This model was, however, a scenario analysis and not a full economic evaluation. Furthermore, it did not focus on a specific programme, but rather a range of programmes likely to be implemented in the UK.

The current study conducted an economic evaluation of the Triple P programme, one of the best-researched examples of evidence-based parenting interventions for the treatment of CD in children. Using the Australian population as an example, a health sector perspective was adopted to conduct a cost–utility analysis comparing Triple P with a ‘no intervention’ scenario through the use of modelling techniques.

## Methods

### Economic evaluation framework

This study used a standardized economic evaluation modelling framework developed for evaluating health care interventions in the Australian context [20]. The following principles underpinned the evaluation: (1) the adoption of a health sector perspective, with a focus on government as a third-party payer; (2) costs were divided into those accruing to the government and those accruing to individuals and their families; (3) data on intervention effectiveness were sourced from published literature; (4) a cost–utility analysis was performed using disability-adjusted life years (DALYs) averted as the primary outcome; (5) a “partial null” comparator was chosen to represent the theoretical level of disease that would be present if no interventions for this disease were in place [21]; (6) the model used a 2013 reference year to match the latest Global Burden of Disease (GBD) study [22]; (7) costs were measured in 2013 Australian dollars; (8) a 3% annual discount rate was applied to both costs and health outcomes; and (9) we adopted a threshold value for cost-effectiveness of $50,000 per DALY, a commonly accepted value for money threshold in the Australian setting [20, 23].

### Literature search

To find evidence on the effectiveness of Triple P, we performed a search of existing reviews alongside a supplementary search of additional studies. The inclusion criteria were: (1) randomized controlled trials or quasi-experimental designs; (2) interventions for the treatment of CD (targeting children with a diagnosis of CD based on structured clinical interviews or cutoffs from disease-specific symptom rating scales); (3) studies reporting diagnostic/clinical outcomes at follow-up; and (4) interventions that are currently used for the treatment of diagnosed CD and would optimally operate within the Australian mental health system (based on advice from a Technical Advisory Panel composed of clinical experts and researchers in the field). This study modelled changes in disorder prevalence that would occur with the Triple P intervention. As such, we excluded studies measuring changes in CD symptoms (without reporting clinical cutoffs) due to difficulties in determining how changes in a mean and standard deviation score on a symptom scale translate into actual cases of CD. Furthermore, the most recent GBD study has one weight for CD, therefore, making it impossible to meaningfully model changes in severity levels, which failed to achieve clinical remission [1]. A complete description of the search strategy is presented in Section 2 of the Supplementary Appendix.

### Intervention effect sizes

From the literature search, the majority of studies were on the effectiveness of level 4 Triple P. Of these, two studies fulfilled our inclusion criteria [24, 25]. Thus, the group [24] and the individual [25] face-to-face formats were selected for economic evaluation. Martin and Sanders [24] assessed the effectiveness of group Triple P targeting 2–9 year olds, while Sanders et al. [25] assessed the effectiveness of individual Triple P targeting 3 year olds. Both studies reported outcomes at post-test (follow-up time ranged between 8 weeks after baseline for the group format to 10 weeks after baseline for the individual format). Children were assessed for conduct problems prior to randomization via a telephone consultation. Both studies used the Eyberg Child Behaviour Inventory (ECBI) as the outcome measure instrument [26], and included children who scored within the clinical range of the ECBI intensity scale completed by parents. Group Triple P included four weekly two-hour group sessions delivered by two psychologists, followed by four weekly individual telephone consultations with an average duration of 30 min. Parents were also given a workbook. Individual Triple P included ten individual sessions, lasting between 60 and 90 min, delivered by one psychologist.

We calculated an effect size for each intervention at post-test, expressed as a relative risk (RR) with a 95% confidence interval (CI). This resulted in a relative risk (RR) of: 0.054 (95% CI 0.003–0.875) for group Triple P and 0.655 (95% CI 0.484–0.887) for individual Triple P. We assumed that intervention effects at post-test would be maintained up to 1-year follow-up, as the literature supports the impacts of many parenting interventions remain up to 1 year [12, 17, 27, 28]. A null effect size was assumed thereafter given the lack of published evidence on the sustainability of effects beyond this period. We assumed that study completers would receive a full intervention effect, while dropouts would incur a cost, but receive no benefit. In clinical work with children, 40–60% drop out of treatment prematurely and hence may not be receiving the benefits of treatment [29]. We defined dropouts as parents who completed less than 20% of the intervention [29–31] (Table 1).

**Table 1:** Input parameters and uncertainty ranges used to model health benefits

Parameters	Value and uncertainty range	Distribution used in PSA	Sources
Patient flowchart			
Proportion of parents offered the intervention	60% (range 40–80)	Pert	Consultation with TAP
Proportion of parents taking up the intervention	60% (range 40–80)	Pert	NSMHWB [32]
Proportion dropouts (completing 20% of intervention (2 sessions)	42% (range 36–49)	Pert	1-proportion of completers [29–31]^a^
Proportion of parents completing the intervention	58% (range 52–65)	Pert	Own meta-analysis of studies [29–31]^a^
Epidemiological inputs			
Population	5–9-year-old children with CD		2013 Australian population [38]
All-cause mortality	Single age rates		2013 Australian life tables [39]
Prevalence of conduct disorder	Average 5–18 age range 0.026 (see Supplementary Appendix for age- and sex-specific estimates)	Beta	[22]
Remission	Average 5–18 age range 0.304 (see Supplementary Appendix for age- and sex-specific estimates)	Gamma	[22]
Incidence	Average 5–18 age range 0,009 (see Supplementary Appendix for age- and sex-specific estimates)	Gamma	[22]
Case fatality	0^b^		[22]
Disability weight for conduct disorder	0.241 (95% CI 0.159–0.341)^c^	Beta	[22]
Effect size group Triple P	0.054 (95% CI 0.003–0.875)^d^	Lognormal	[24]
Effect size individual Triple P	0.655 (95% CI 0.484–0.887)^d^	Lognormal	[25]

*PSA* probabilistic sensitivity analysis, *TAP* technical advisory panel, *NSMHWB* 2014 National Survey of Mental Health and Well-Being—child component, *CI* confidence interval

^a^See Section 3 of the Supplementary Appendix for detailed methods on the calculation of the proportion of intervention completers and dropouts

^b^As per the 2013 GBD study, case fatality was zero. This is because no estimate of excess mortality due to CD was found in the literature

^c^Unadjusted disability weight used as the cohort of children modelled in this study was symptomatic and determined appropriate for intervention; therefore, the “weighted” disability weight reported in [22] is an underestimate, given it includes children with a less severe condition

^d^A follow-up study of Sanders et al. [27] reports the outcomes of individual Triple P at 1- and 3-year follow-up (the control group is dropped after post-test) and treatment gains are maintained at both follow-up periods. The initial effect size of the intervention targeting 3 year olds was assumed to remain until 6 years of age

### Study population and intervention pathway

This study modelled the delivery of group and individual Triple P targeting children aged 5–9 years with CD in the 2013 Australian population. We limited the scope of the model to children with CD currently seeking treatment, because the intervention does not include a new case finding component, but rather targets parents of children with CD who are treatment seeking. This was to reflect current practice, since only 60% of children with CD in Australia currently access treatment [32]. The selected age group reflects the ages comprised in the trials used to provide the effectiveness estimates. Although the trial on individual Triple P targeted children younger than 5 years, our model applied a lower age limit of 5 years, as this reflects the age at which a diagnosis of CD can be given in clinical practice.

We developed an intervention pathway that was representative of routine health services delivered within the Australian mental health care system. We assumed the following intervention pathway: (1) children with conduct problems attended a first visit with a general practitioner (GP) who performed an initial assessment prior to making a referral to a psychologist; (2) parents were offered either group or individual Triple P; and (3) upon treatment completion, the child was called for at least one follow-up visit with the GP. The following parameters were considered when calculating the eligible population that participated in each intervention: (a) proportion of children with a CD diagnosis; (b) proportion of parents attending an initial GP visit and offered the intervention; (c) proportion of parents taking up the intervention; (d) proportion of parents dropping out; and (e) proportion of parents completing the intervention. We assumed one parent per child. The eligible population was partitioned by sex and 1-year age groups. All parameters, assumptions, and data sources are shown in Table 1 with the patient flowchart presented in Section 3 of the Supplementary Appendix.

### Modelling health benefits

A population-based multiple cohort decision analytic model with 1-year cycles was implemented in Excel to simulate the disease dynamics with and without the delivery of either group or individual therapy over a 13-year time horizon. An adapted Dismod-II model, based on a set of differential equations that describe age-specific epidemiological parameters, was used to simulate how a population cohort moves between three health states over time—i.e., healthy, diseased, and dead [33]. The diseased health state included all children with CD, with the prevalence of CD in the initial cycle (i.e., at year 0) being based on the current prevalence of CD in the 2013 Australian population for each respective age–sex cohort (see Section 4 of the Supplementary Appendix for the corresponding state transition diagram). Transitions between health states corresponded with the epidemiological parameters: incidence, remission, case fatality, and all-cause mortality. The model calculated annual transitions between health states for each single year of age–sex cohorts starting at age 5 until age 9, until children reached adulthood (i.e., 18 years). CD was modelled along the lines of a chronic, rather than an episodic disorder. Children were assigned a single disability weight for CD (as per the GBD studies [1, 34]) based on the presence or the absence of the disorder. Comorbidities and longer term consequences related to CD were not modelled, as there is no literature supporting the longer term effectiveness of Triple P in reducing CD or related comorbidities.

Aggregated health outcomes were expressed as DALYs, using the disability weight (DW) for CD (0.241) from the GBD [34] and the prevalence of CD. Only the morbidity component of the DALY was modelled in our study, as there was no evidence of a mortality impact. The model produced incremental cost-effectiveness ratios (ICERs) reported as the net cost per DALY averted. A summary of the epidemiological inputs and their sources are shown in Table 1, and detailed methods described in Section 4 of the Supplementary Appendix.

### Costing analysis

The costs of delivering the intervention included: the cost of psychological assessment by a GP; the cost of a practitioner to deliver group and individual sessions and telephone consultations; and the cost of the workbooks. We assumed that the interventions would be delivered through the publicly-financed community sector. Occasionally, participants were charged out-of-pocket costs, depending on the type of service and the provider. We assumed that the costs for psychological assessment by a GP were fully borne by the government. By contrast, Medicare Benefits Schedule (MBS) items for GP consults and group/individual psychologist visits were split between: the government (who paid 85% of the listed MBS item fee) and the parents (who paid the remaining 15% out-of-pocket). We assumed that the cost of programme materials was borne by the parents.

Time and travel costs accruing to parents were excluded from the base-case analysis, but included in a sensitivity analysis. Cost offsets (i.e., treatment costs that are avoided due to the reduction in the prevalence of CD), which accrue to the health sector, were included in the base-case analysis. Broader societal perspectives deemed relevant were included in the sensitivity analysis, as large costs associated with CD fall outside the health care sector (e.g., criminal justice).

The model assumed that the interventions were fully implemented and operating under “steady state” conditions—i.e., trained staff and necessary infrastructure were available to deliver the intervention, which operated in accordance with its effectiveness potential [35]. A full description of the methods used in the costing analysis are presented in Section 5 of the Supplementary Appendix. All inputs and sources used in the costing analysis are shown in Table 2.

**Table 2:** Input parameters and uncertainty ranges used in the model for costing analysis

Cost parameters (AUS$)	Value and uncertainty range	Distribution used in PSA^c^	Sources
Cost of general practitioner (first visit)	Government: $97.27		MBS items 2700, 2701, 2715 and 2717 [40]
Cost of general practitioner (follow-up visit)	Government: $70.30		MBS item 2712 [40]
Cost of MBS-funded psychologist single	Government: $102.34; private: $18.06		MBS items 80,010 and 80,110 [40]
Cost of MBS-funded psychologist group	Government: $25.61; private: $4.52		MBS items 80,020 and 80,120 [40]
Cost workbooks Triple P			Triple P practitioner resources^a^
Every parent	Private: $35		
Every parent group workbook	Private: $14.95		
Cost uncertainty parameter out-of-pocket costs	Range ± 20% of unit costs	Pert	Protocol
Annual cost of a prevalent case of conduct disorder (for the calculation of cost offsets)			
Health care	5–10 years: $1076.03 (both males and females) (range ± 20%)	Pert	Own calculations (see Section 5.2 of the Supplementary Appendix)
	11–18 years: males: $333.01; females: $141.03 (range ± 20%)	Pert	Own calculations (see Section 5.2 of the Supplementary Appendix)
Other sector costs^b^	5–10 years: $2270.85 (both males and females) (range ± 20%)	Pert	Own calculations (see Section 5.2 of the Supplementary Appendix)
	11–18 years: males: $10,854.29; females: $1027.68 (range ± 20%)	Pert	Own calculations (see Section 5.2 of the Supplementary Appendix)
Time cost (per hour)^b^	$9.96		(Uprated to 2013 AU$) [35]
Travel cost (per trip)^b^	$24.67		(Uprated to 2013 AU$) [35]
Discount rate	3%		[35]

*MBS* Medicare Benefits Schedule

^a^ http://www29.triplep.net/files/pdf/TripleP_Australian_Order_Form_for_PRACTITIONERS.pdf

^b^Time and travel costs and other sector costs were not included in the base-case analysis, only used in the sensitivity analysis

^c^A Pert ± 20% distribution was used for every cost parameter which had uncertainty modelled

### Uncertainty and sensitivity analyses

ICERs were calculated by dividing the estimated difference in costs by the estimated difference in DALYs averted through the decrease in prevalence of CD for each intervention compared to “no intervention”. We used Ersatz (version 1.31, Sunrise Beach, Australia, available at: http://www.epigear.com/) to perform a probabilistic uncertainty analysis, using Monte Carlo simulation with 3000 iterations to produce 95% uncertainty intervals (95% UIs) around the DALYs averted, net costs, and ICERs. Uncertainty parameters are shown in Tables 1 and 2.

Univariate sensitivity analyses were performed to investigate the impact of specific input parameters and assumptions on the model outcomes. We modelled the individual impact of: (1) excluding cost offsets related to the health care sector; (2) including cost offsets related to both the health care sector and other non-health sectors; (3) including time and travel costs; (4a) assuming a 50% decay rate in effects after year 1 over 5 years (by year 5 the RR is close to the null); (4b) assuming intervention effects persist over 5 years; (5) assuming dropouts get half of the benefit; and (6) applying a 0 and 6% discount rate to both costs and benefits. We also conducted a threshold analysis to test the impact of varying the effect size of both group and individual Triple P.

## Results

**Fig. 1:**
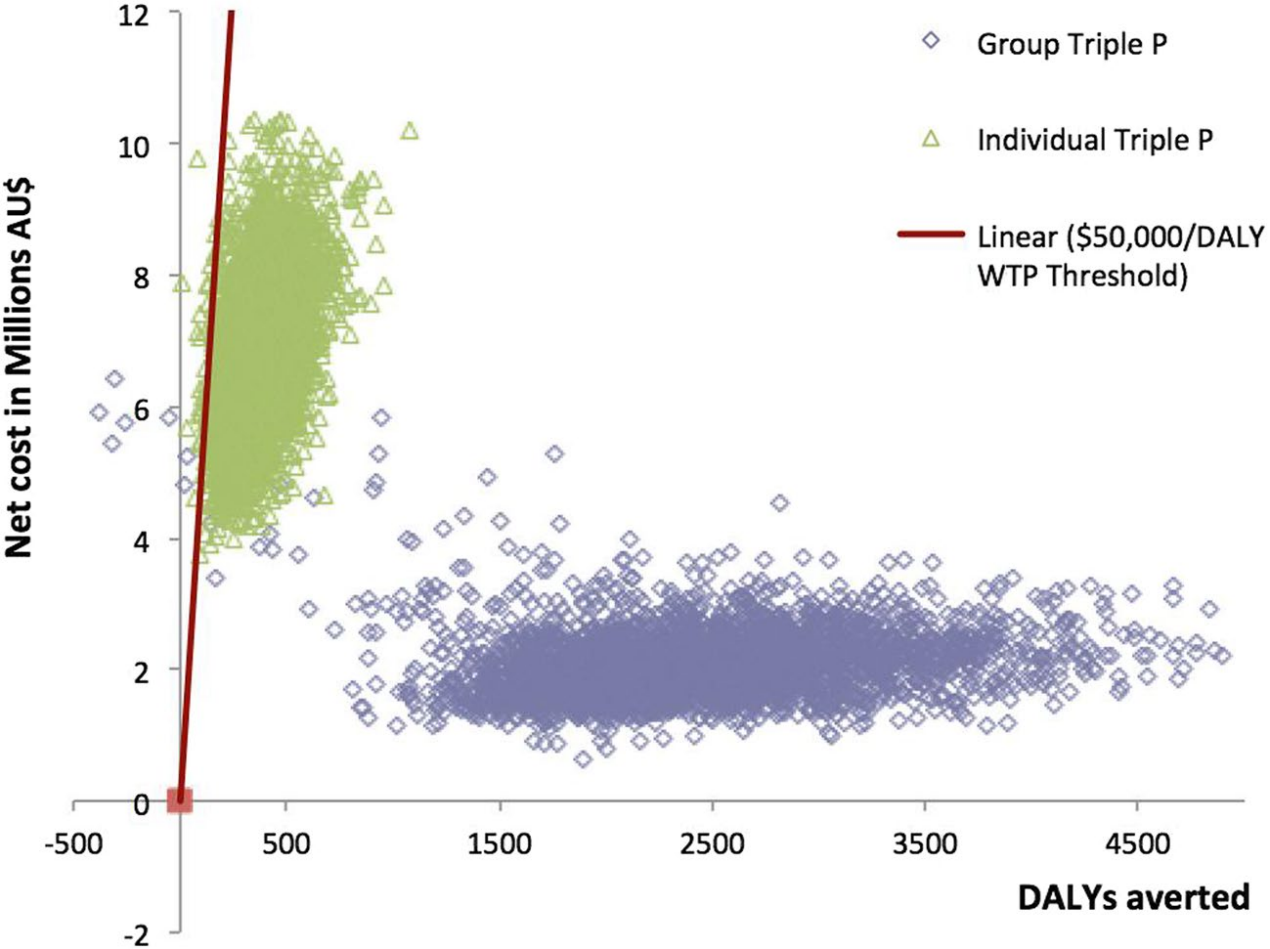
Cost-effectiveness plane of the base-case analysis

**Fig. 2:**
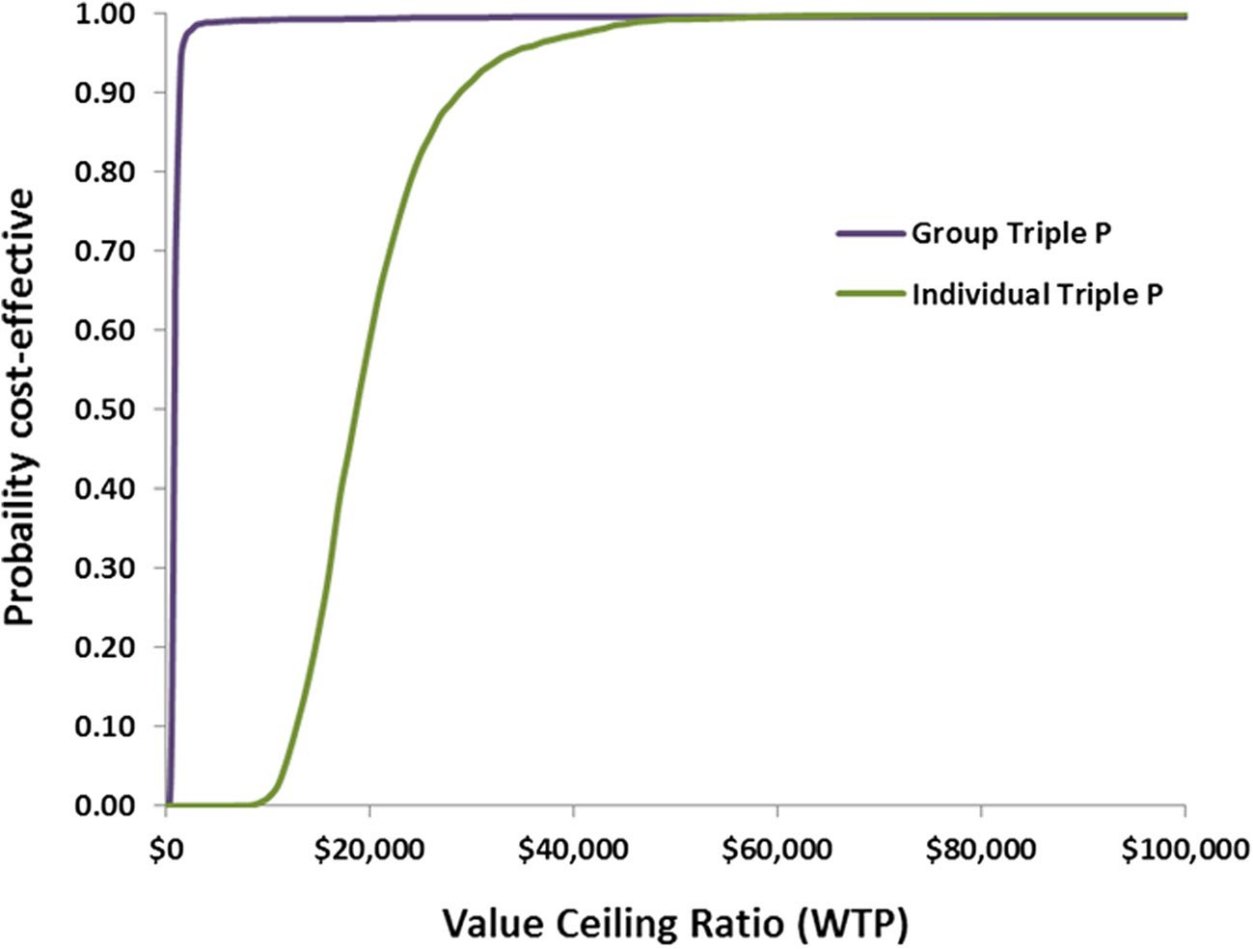
Cost-effectiveness acceptability curve of the base-case analysis

Table 3 shows the base-case cost-effectiveness results. Group Triple P was very cost-effective relative to a threshold of $50,000 per DALY averted, with an ICER of $1013. Individual Triple P was cost-effective, with an ICER of $20,498 per DALY averted. Figure 1 presents the results of the base-case analysis on a cost-effectiveness plane, where estimated cost differences are plotted against estimated differences in DALYs averted between the intervention and “no intervention”. In this figure, we can see that the uncertainty iterations for both interventions lie on the north-east quadrant of the plane, where the intervention is more effective and more costly than the comparator. Furthermore, the majority of the iterations fall below the cost-effectiveness threshold of $50,000 per DALY averted. The cost-effectiveness acceptability curve (Fig. 2) shows the probability of group and individual being cost-effective for a range of willingness-to-pay (WTP) values a decision maker would be willing to pay per additional DALY averted. It is evidenced that the probability that both intervention formats are cost-effective at a threshold of $50,000 per DALY averted is close to one (appx. 99%), with group Triple P having a high probability of cost-effectiveness already at very low values of WTP.

**Table 3:** Results of the base-case model examining the cost-effectiveness of group and individual Level 4 Triple P

Intervention delivery format	Mean ICER (95% UI) (AU$/DALY averted)	DALYs averted (95% UI)	Intervention costs (AU$) (95% UI)			Cost offsets (AU$) (95% UI)	Net costs (AU$) (95% UI)
			Government	Private	Total		
Group	1013 (471–1956)	2421 (1234–4116)	4.6 M (3.3–6.1 M)	912,862 (610,381–1.3 M)	5.5 M (3.9–7.4 M)	3.4 M (2 M–5 M)	2.1 M (1.3–3.4 M)
Individual	20,498 (11,146–39,470)	371 (161–670)	6.3 M (4.4–8.4 M)	859,465 (567,906–1.2 M)	7.2 M (5–9.6 M)	333,236 (163,830–554,751)	6.8 M (4.8–9.2 M)

*M* millions

**Fig. 3:**
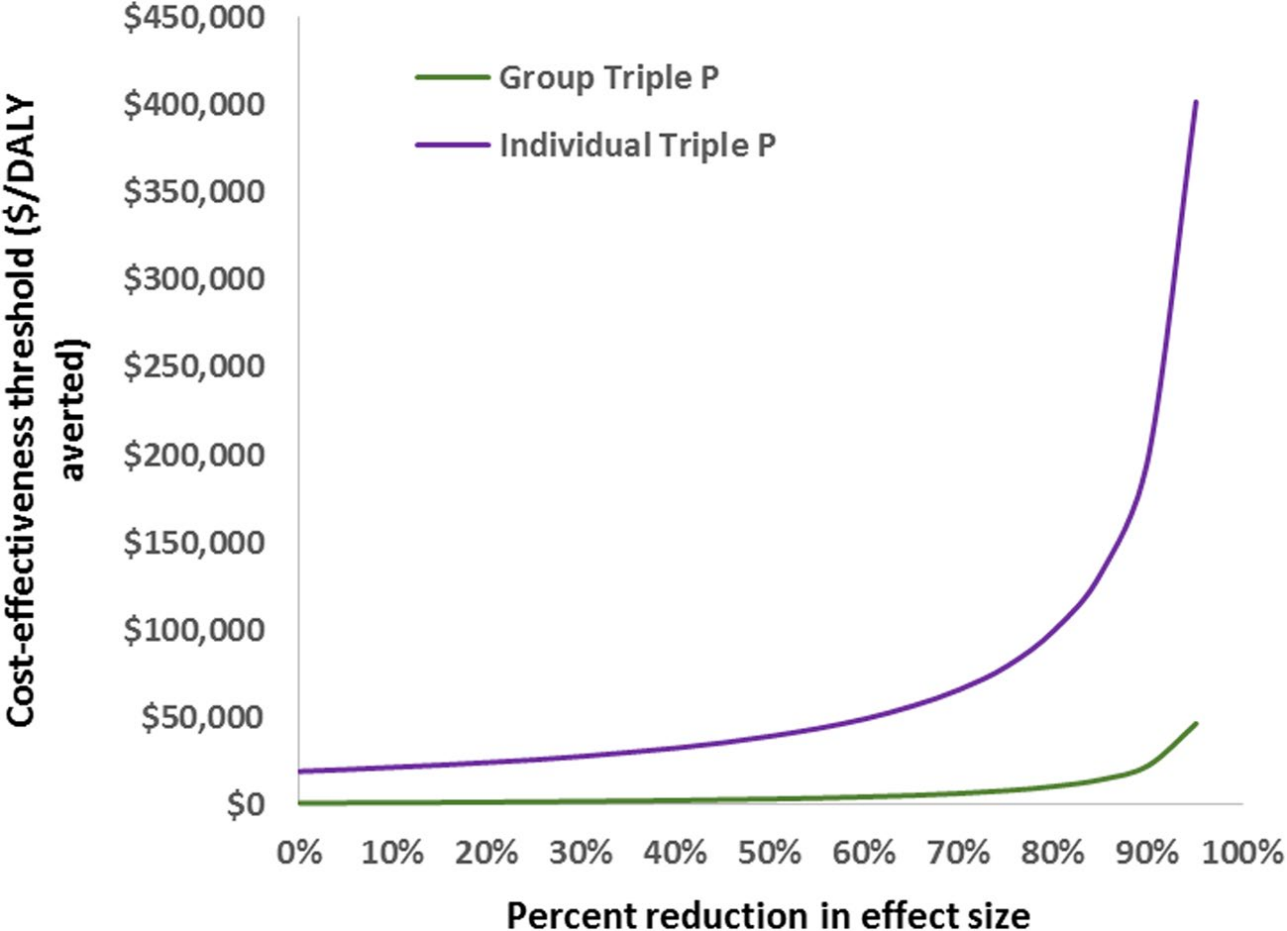
Threshold analysis to examine the impact of varying the effect size

The results of the sensitivity analyses are presented in Table 4. The cost-effectiveness results for both interventions differed little from the base-case when: assuming differential discount rates and assuming dropouts get 50% of the health benefits. The cost-effectiveness of both interventions improved greatly when assuming intervention effects persisted over 5 years, with group Triple P becoming cost saving. The exclusion of cost offsets pertaining to the health sector led to slightly higher ICERs for both interventions compared with the base-case. The inclusion of time and travel costs (total time and travel costs: $2.5 M group; $2.8 M individual) tripled the ICER for group Triple P, whereas it led to individual Triple P having a slightly higher ICER and a lower probability of being cost-effective. Group Triple P became cost saving when including cost offsets pertaining to all sectors (cost offsets: $10.5 M group; $1 M individual), whereas the ICER for individual Triple P decreased marginally. Assuming a 50% annual decay rate of the intervention effect led to group Triple P becoming cost saving, but with a wide uncertainty interval which intersected the south-east, north-east, and north-west quadrants of the plane—i.e., being either cost saving (dominant) or economically inefficient (dominated). This scenario also resulted in individual Triple P being more cost-effective, but with a wide uncertainty interval spanning over both the north-west and the north-east quadrants of the plane—i.e., with a double likelihood of being less effective and more costly than the comparator (dominated), and more costly and more effective than the comparator. In the threshold analysis (Fig. 3), group Triple P remained cost-effective to the threshold of $50,000 per DALY averted despite variations in effect. Individual Triple P remained below the threshold assuming an effect size 60% lower than the base-case.

**Table 4:** Results of univariate sensitivity analysis for the base-case model examining the cost-effectiveness of group and individual level 4 Triple P

Sensitivity analysis	Group ICER (95% UI) (AU$/DALY averted)	Individual ICER (95% UI) (AU$/DALY averted)
Base-case analysis	1013 (471–1956)	20,498 (11,146–39,470)
(1) No cost offsets (health care)	2460 (1542–3871)	21,430 (11,828–40,682)
(2) With cost offsets (health care + other sector costs)	Dominant^c^	18,527 (9564–37,207)
(3) With time and travel costs	3567 (2233–5624)	29,903 (16,532–56,903)
(4a) 50% decay in effect size over 5 years	Dominant^c^ (dominant to 2117)^a^	13,911 (dominated^d^ to 77,650)^b^
(4b) Full effect size extrapolated over 5 years	Dominant^c^	2336 (1306–4009)
(5) Dropouts get 50% of health benefit	927 (477–1889)	20,248 (10,756–38,509)
(6a) Discount rate of 0%	955 (439–1779)	19,820 (10,610–38,218)
(6b) Discount rate of 6%	980 (494–2211)	20,894 (11,295–41,068)

^a^A proportion of the uncertainty iterations lie in the south-east, north-east, and the north-west quadrants of the cost-effectiveness plane, signifying that there is a likelihood that the intervention is more effective and more costly than the comparator, that it is less costly and more effective than the comparator (dominant), and that it is more costly and less effective than the comparator (dominated)

^b^A proportion of the uncertainty iterations lie in both the north-west and the north-east quadrants of the cost-effectiveness plane, signifying that there is a likelihood that the intervention is less effective and more costly than the comparator (dominated) and that it is more costly and more effective than the comparator

^c^The intervention is less costly and more effective than the comparator (dominant)

^d^The intervention is less effective and more costly than the comparator (dominated)

Further details on the sensitivity analyses are presented in Section 6 of the Supplementary Appendix.

## Discussion

### Main findings and comparison with other studies

Using the Australian population as an example, this study used a health care perspective to investigate the cost-effectiveness of a well-known evidence-based parenting intervention, group and individual face-to-face Triple P, compared to no intervention, for the treatment of CD in children. This study demonstrated that both intervention formats are good value for money, with the group face-to-face format having a higher probability of cost-effectiveness. Univariate sensitivity analyses conducted corroborated the results, demonstrating that the interventions remained cost-effective when making different assumptions about modelling parameters. The biggest impact was that of varying intervention effectiveness estimates. Group Triple P remained cost-effective despite variations in effect, whereas the cost-effectiveness ratio for individual Triple P went above the threshold of $50,000 per DALY when assuming an effect size 60% lower than the base-case.

This is the first study evaluating the longer term cost-effectiveness of parenting programmes in general and of Triple P in particular for the treatment of CD using a cost–utility framework. Bonin et al. [19] estimated the costs and longer term savings of parenting programmes in the UK associated with the reduction in the probability of persistent CD among children. They modelled a “generic” parenting intervention, based on various evidence-based programmes that were likely to be implemented in the UK. Their study demonstrated that parenting programmes were likely to reduce the probability of CD persisting into adulthood, as well as they would entail savings to the public sector in the short run, yielding about US$27,136 per family over 25 years. These findings cannot, however, be compared to the findings of the current study, as the study from Bonin et al. [19] was not a full economic evaluation, but rather, a cost-offset scenario analysis where inputs were varied according to three intervention scenarios (base, best, and worst cases). Another similar study from Mihalopoulos et al. [18] modelled the expected long-term costs and cost savings of implementing different intensity levels of Triple P (levels 1–5) at a population level. The study concluded that Triple P had the potential to be cost saving over the long-term if at least 7% of cases of CD were averted. Although not directly comparable, both studies conducted to date demonstrate the potential of Triple P being cost saving in the long run.

### Implications for policy and practice

Clinically, while group Triple P is very cost-effective, it is not always possible or appropriate to provide group-based therapies for all families due to, for instance, limited facilities, trained staff or treatment specifications per se. In this instance, it is still cost-effective to provide individual-based Triple P, although in terms of value for health care, group-based therapy should be the preferred mode of delivery, where possible.

In terms of generalizability of this study’s results, a few issues need to be considered. Although the context of the study was Australian, the methods and results have international relevance. Triple P was developed in Australia, but is implemented in various countries; thus, while referral pathways can differ between countries, the intervention itself would not differ. In our model, we assumed that the interventions, which were the most advanced forms of Triple P, were delivered by psychologists via the publicly-funded Medicare system. This was to reflect the common practice within Australian mental health services, as well as the main professional category likely to deliver this level of Triple P. It is, however, important that readers bear in mind that the cost-effectiveness results may be different in other settings, mostly due to differences between health care systems in terms of structure, financing, price levels, and service provision. Thus, results should be generalized only to countries with similar health care systems to the Australian one, such as the Swedish and the British one, where these interventions would likely be funded publicly. Conversely, the results could dramatically change in contexts such as the United States, where such interventions are likely to be funded privately.

In addition, in this study, only Triple P was considered (although other parenting programmes have similar effect sizes measured using symptom scales), with the purpose of model testing, due to its well-established implementation in various international settings. Nevertheless, this model is flexible and can be used for economic evaluations of other interventions targeting CD in the Australian setting, and in other international settings, and thus assist priority setting.

### Strengths and limitations

To the authors’ knowledge, this is the first study to assess the cost-effectiveness of Triple P using a cost–utility population-based economic modelling approach. Other strengths are the use of the latest data on CD epidemiology; the use of a standardized evaluation framework to avoid methodological confounding, and ensure comparability and transparency of results; and applying conservative assumptions where possible.

There are a few challenges to this kind of research. The estimates were sourced from one study each due to the limited amount of studies fulfilling our inclusion criteria. Given that the therapeutic content of group and individual Triple P is similar in terms of quantity of therapeutic time but not in terms of content, we would have expected individual therapy to be more effective than group therapy. This large difference in effect size is probably due to the disparity in study population characteristics (i.e., the study on group Triple P drew participants from academic and general staff in a university setting [24]), whereas the study on individual Triple P targeted low-income areas with high rates of unemployment, where eligible families had at least one adversity factor, such as maternal depression or single parenthood [25]. Families with more adversity factors have been shown to be less likely to respond to treatment [36]. With this in mind, the cost-effectiveness of individual Triple P may have been underestimated and that of group Triple P overestimated.

Although the studies used to source the effectiveness estimates only demonstrate the effects of Triple P remain up to 8–10 weeks, our base-case results support the cost-effectiveness of Triple P for the treatment of CD delivered within the Australian healthcare setting.

Another issue pertains to the use of symptom-based scales to determine clinical caseness and how well they reflect the true impacts of an intervention. Despite the ECBI being a good predictor of CD, using the cutoff as a measure of clinical caseness may underestimate the full potential impacts of the intervention. Impacts on children who had, at start, higher scores, but did not make it through the cutoff would not be captured. We have, however, used all best available evidence, with this limitation in mind.

Importantly, although DALYs are acceptable to Australian decision makers [20], its use as an outcome measure poses limitations. The most substantial problem is the availability of a single disease weight. This makes it impossible to model changes in disease severity without making sweeping assumptions about the distribution of disease and the disability weight by severity [34] (CD does not have differential severity weights; for example, depression has differential weights for mild, moderate, and severe diseases). Consequently, the present study only modelled impacts of the interventions on diagnosed children. The study did not include children with sub-threshold levels of CD or with some levels of problems, who may also benefit from this intervention. Therefore, there is likely to be a substantial underestimation of health gains attributable to reductions in severity. A better approach should specify weights based on disease severity to reflect heterogeneity in health [37].

It is also important to appreciate that the current study only included impacts of the interventions on the children themselves, excluding any benefits that may incur to those directly affected by CD, such as parents, siblings, teachers, and peers. A parenting intervention that successfully improves CD may also reduce caregiver burden and improve the relationship of the child with the parents and with others in the near social circle, which will have impacts on the quality-of-life of these individuals. Impacts on parents are evidenced in the literature demonstrating that parenting interventions have a positive impact on parenting skills and parental mental well-being [11, 16]. It is thus important that economic evaluation includes impacts on all relevant individuals affected by parenting interventions, so that appropriate decisions can be made. Further research may be needed to consider this issue.

## Conclusions

Evidence-based parenting programmes, such as the Triple P, for the treatment of CD in children, appear to represent good value for money, when delivered in both group and individual face-to-face formats, with the group format being the most cost-effective option. The results should, however, be interpreted with caution, as there are several limitations, including: the insufficient quality of the evidence for the interventions modelled and the restrictive inclusion of diagnosed cases of CD as the main outcome measure. Furthermore, the current model can be used for economic evaluations of other interventions targeting CD and in other settings.

## Electronic supplementary material

Below is the link to the electronic supplementary material.

Supplementary material 1 (DOCX 390 kb)
