# Supplementary material for: Population cost-effectiveness of the Triple P parenting programme for the treatment of conduct disorder: an economic modelling study
Source: Eur Child Adolesc Psychiatry. 2017 Dec 29;27(7):933–44. doi: 10.1007/s00787-017-1100-1 (PMC6013530; doi:10.1007/s00787-017-1100-1)
Supplement: Supplementary file 1 — Supplementary material 1 (DOCX 390 kb) [file 787_2017_1100_MOESM1_ESM.docx]

Supplementary appendix

Population cost-effectiveness of the Triple P parenting programme for the treatment of Conduct Disorder: an economic modelling study

Filipa Sampaio^1^, Jan J Barendregt^2,3^, Inna Feldman^1^, Yong Yi Lee^3,4^, Michael G Sawyer^5,6^, Mark R Dadds^7^, James G. Scott^4,8,9^, Cathrine Mihalopoulos^10^

**Affiliations:**

^1^Department of Public Health and Caring Sciences, Uppsala University, Uppsala Sweden; ^2^Epigear International, Sunrise Beach, Queensland, Australia;

^3^School of Public Health, The University of Queensland, Herston, Queensland, Australia; ^4^Queensland Centre for Mental Health Research (QCMHR), The Park Centre for Mental Health, Wacol, Queensland, Australia;

^5^School of Medicine, University of Adelaide, Adelaide, South Australia;

^6^Research and Evaluation Unit, Women's and Children's Health Network, Adelaide, South Australia, Australia;

^7^Child Behaviour Research Clinic, University of Sydney, Sydney, Australia;

- ^8^The University of Queensland Centre for Clinical Research, Herston, Queensland;
- ^9^Metro North Mental Health, Royal Brisbane and Women's Hospital, Herston, Queensland;

^10^Deakin Health Economics, School of Health and Social Development, Deakin University, Melbourne, Australia

**Address correspondence to:** Filipa Sampaio, Department of Public Health and Caring Sciences (IFV), Uppsala University, BMC, Husargatan 3, 751 22 Uppsala

Sweden, [filipa.sampaio@pubcare.uu.se](mailto:filipa.sampaio@pubcare.uu.se), +46 (0)18-471 65 61

**E-mail addresses:**

Jan J Barendregt - j.barendregt@sph.uq.edu.au

Inna Feldman – inna.feldman@pubcare.uu.se

Yong Yi Lee - y.lee5@uq.edu.au

Michael G Sawyer - michael.sawyer@adelaide.edu.au

Mark R Dadds - mark.dadds@sydney.edu.au

James G. Scott - james.Scott@health.qld.gov.au

Cathrine Mihalopoulos - cathy.mihalopoulos@deakin.edu.au

Table of Contents

1. Overview of the work 3

2. Intervention evaluated and effect sizes 3

2.1. Intervention description 3

2.2 Literature search 3

2.3 Literature search results 4

2.4 Intervention effect sizes 7

3. Identification of the eligible population 7

4. Modelling health benefits 11

4.1. Model structure 11

4.2. Model parameters 13

4.2.1 Incidence 13

4.2.2 Remission 13

4.2.3 Case fatality 14

4.2.4 All-cause mortality 14

4.2.5 Prevalence 15

4.2.6 Disability weight 15

5. Cost analysis 15

5.1. Cost analysis of group and individual Triple P 15

5.2. Cost offsets 20

6. Sensitivity analyses 20

6.1. Sensitivity analysis excluding cost offsets related to the health care sector 21

6.2. Sensitivity analysis including cost offsets related to both the health care sector and other non-health sectors 21

6.3. Sensitivity analysis including time and travel costs 21

6.4. Sensitivity analysis assuming a 50% decay rate in effect sizes after year 1 over 5 years 23

6.5. Sensitivity analysis assuming intervention effects persist over 5 years 23

6.6. Sensitivity analysis assuming dropouts get half of the benefit (50% of the effect size 23

6.7. Sensitivity analysis applying different discount rates to costs and benefits 24

7. Consolidated Health Economic Evaluation Reporting Standards (CHEERS) statement 25

7. References 30

# 1. Overview of the work

This document aims to provide a complete description of the methods used in the study entitled: “Population cost-effectiveness of the Triple P parenting programme for the treatment of Conduct Disorder: an economic modelling study”. In this study we developed a decision-analytic model for Conduct Disorder (CD), which can be used to model the population cost-effectiveness of various preventive and treatment interventions. For the first run of the model the cost-effectiveness of the Triple P parenting programme for the treatment of CD in children and adolescents in Australia was evaluated. Triple P was chosen, as this is one of the best researched examples of evidence-based parenting interventions for the treatment of CD in children with strong Australian efficacy credentials.

# 2. Intervention evaluated and effect sizes

## 2.1. Intervention description

While many parenting interventions exist, one of the most widely researched and internationally disseminated programmes is the Triple P - Positive Parenting Programme [[1](#_ENREF_1)]. Triple P is a behavioural family intervention aimed at preventing and treating severe behavioural, emotional and developmental problems in children and adolescents by enhancing the knowledge, skills and confidence of parents. The programme incorporates five levels of intervention of increasing intensity and narrowing population reach for parents of children from birth to age 16. It comprises both universal and targeted interventions designed to create a family-friendly environment that better supports parents in the task of raising their children, with a range of problems tailored to the different needs of parents. The five levels of intervention vary according to intensity, contact with practitioners, and delivery format. Level 1 is a media and communication strategy on positive parenting (e.g., television, radio, online, and print media); level 2 includes brief interventions consisting between one or three sessions (e.g., telephone or face-to-face or group seminars); level 3 consists of narrow-focused interventions including three to four individual face-to-face or telephone sessions, or a series of 2-hour group discussion sessions; level 4 includes 8-10 sessions delivered through individual, group or self-directed (online or workbook) formats; and level 5 includes enhanced interventions using adjunct individual or group sessions addressing additional problems [[2](#_ENREF_2)].

## 2.2 Literature search

For the purposes of the decision analytic model, we opted to use the strongest level of evidence designated by the Australian National Health and Medical Research Council [[3](#_ENREF_3)], i.e. systematic reviews of randomized controlled trials. To find evidence on the effectiveness of Triple P, we performed a literature search of existing reviews using the electronic databases EBSCOhost, INFORMIT Health Collection, SCOPUS and Google advanced search with the following terms: ((Triple P OR Positive Parenting Program*) AND (Conduct disorder*) AND (effective* OR evaluat*)). We also performed an additional search of studies conducted between January 2014 and October 2015 to look for recently published studies that may have not been included in the most recent review of Triple P studies published in early 2014 [[4](#_ENREF_4)].

We selected studies from the reviews and additional studies that fulfilled the following inclusion criteria: (1) randomized controlled trials or quasi-experimental designs; (2) interventions for the treatment of CD (targeting a group of children with a diagnosis of CD based on structured clinical interviews or cut-offs from disease-specific symptom rating scales); and (3) studies reporting diagnostic/clinical outcomes at follow-up (i.e., the number of prevalent cases measured using structured clinical interviews or disease-specific symptom rating scales with good predictive value of CD according to published literature). Given the context of our study is to estimate the avertable disease burden (as measured by the most recent GBD studies [[5](#_ENREF_5), [6](#_ENREF_6)]) we focussed on diagnosed disorder.

This study modelled changes in disorder prevalence that would occur with the Triple P intervention. As such, we excluded studies measuring changes in CD symptoms (without reporting clinical cut-offs) due to difficulties in determining how changes in a mean and standard deviation score on a symptom rating scale translate into actual cases of CD. Furthermore, the most recent GBD study has one weight for CD [[5](#_ENREF_5)]. Therefore, even though changes in symptom severity can be a positive outcome of a disease (without a necessary change in meeting the criteria for a diagnosis), modelling this impact using a single disease weight would require additional assumptions.

## 2.3 Literature search results

We found four reviews in the initial search including studies that examine the effectiveness of Triple P [[4](#_ENREF_4), [7-9](#_ENREF_7)], and two new studies in the additional search [[10](#_ENREF_10), [11](#_ENREF_11)]. From the four selected reviews, the majority of studies were on the effectiveness of level 4 Triple P (n=101). There were no evaluations meeting the criteria above for levels 1-3 Triple P (probably because these levels of Triple P are not necessarily targeting clinical populations). Thus, we selected studies of Triple P level 4 that met our inclusion criteria. Six studies satisfied our inclusion criteria [[12-17](#_ENREF_12)]. The two additional studies found in the supplementary search were excluded as one did not have a control group [[11](#_ENREF_11)], and the other was an indicated prevention study including children who were rated over a clinical cut-off on a symptom-based rating scale by at least one parent, thus the sample of children rated by mothers did not correspond to the sample rated by fathers reported in the study [[10](#_ENREF_10)].

In all six selected studies, children were assessed for conduct problems prior to randomization mostly via a telephone consultation. All studies used the Eyberg Child Behaviour Inventory (ECBI) as the main outcome measure instrument. The ECBI is the most commonly used parental report measure of child conduct problems in children aged 2–16 years, and can assess 36 individual problems [[18](#_ENREF_18)]. The ECBI has good predictive value of CD and good internal consistency. It discriminates between clinical and non-clinical conditions, and a specific cut-off score is recommended to indicate the need for treatment.

Children who scored within the clinical range of the ECBI intensity scale completed by parents were included in the studies. In one study [[17](#_ENREF_17)] the diagnosis of CD was confirmed *a posteriori* with DSM-IV criteria via a semi-structured interview. All interventions included in these studies were delivered to parents by psychologists; one was delivered in group face-to-face format, one individual face-to-face (standard), three self-directed (SD), two SD plus telephone assisted, and one Internet SD. In order to decide which format should best be modelled for the current study, the Technical Advisory Panel composed of clinical experts and researchers in the field assessed how the selected interventions would optimally operate within the Australian mental health system. The self-directed formats of level 4 Triple were therefore excluded as neither being relevant nor currently used for treatment of diagnosed CD. Thus, the group [[14](#_ENREF_14)] and the standard individual [[16](#_ENREF_16)] face-to-face formats were selected for economic evaluation.

The following data were extracted from the two selected studies on group and individual level 4 Triple P: study design characteristics, sample information, outcomes for both intervention and control groups at follow-up (i.e. number of cases/non-cases of CD), and information on the intervention. [Martin and Sanders [14]](#_ENREF_14) assessed the effectiveness of group Triple P targeting a cohort of 2-9 year olds, while [Sanders, et al. [16]](#_ENREF_16) assessed the effectiveness of individual Triple P targeting a cohort of 3 year olds. Both studies reported outcomes for both intervention and control groups at post-test (the time of follow-up ranged between 8 weeks after baseline for the group format to 10 weeks after baseline for the individual format). The study on group Triple P drew participants from academic and general staff at a university setting [[14](#_ENREF_14)], whereas the study on individual Triple P targeted low-income areas with high rates of unemployment, where eligible families had at least one family adversity factor such as maternal depression, single parenthood or marital conflict [[16](#_ENREF_16)].

Table 1. Study design characteristics of the two RCT studies used to estimate the effect size of group and individual Triple P on children with Conduct disorder

| Study | Country | Sample size | Age  range | Population | Version of Triple P | Programme facilitator | Control group | Follow-up | Diagnostic criteria | ITT used |
| --- | --- | --- | --- | --- | --- | --- | --- | --- | --- | --- |
| [Martin and Sanders [14]](#_ENREF_14) | Australia | 45 | 2-9 | Academic and general staff a university | Group | Psychologists | WC | Post-intervention (8 weeks after baseline) | No | No |
| [Sanders, et al. [16]](#_ENREF_16) | Australia | 305 | 3 | Low-income areas with high rates of unemployment, where eligible families had at least one family adversity factor such as maternal depression, single parenthood or marital conflict | Standard individual | Psychologists/  Psychiatrists | WC | Post-intervention (10 weeks after baseline) | No | No |

**Abbreviations:** WC – Waitlist Control

## 2.4 Intervention effect sizes

We calculated an effect size for each intervention at post-test expressed as a relative risk (RR) with a 95% confidence interval (CI). The effect sizes at post-test were:

Group Triple P: RR = 0.054 [95% CI: 0.003 to 0.875]

Individual Triple P: RR = 0.655 [95% CI: 0.484 to 0.887]*

*A follow-up study of Sanders 2000 [[19](#_ENREF_19)] reports the outcomes of individual Triple P at 1- and 3- years follow-up (the control group is dropped after post-test) and treatment gains are maintained at both follow-up periods. The initial effect size of the intervention targeting 3 year olds is assumed to remain until 6 years of age.

In the base-case analysis, we assumed that intervention effects at post-test would be maintained up to 1-year follow-up. We assumed a null intervention effect size after 1-year follow-up. This assumption was supported from plotting outcomes of other RCT studies on the treatment and prevention of CD that have held a control group beyond post-treatment. Study outcomes were plotted and showed that both intervention and control improve throughout time and the difference in effect between conditions seems to be maintained over time.

This assumption is consistent with the literature on the effectiveness of Triple P and other parenting programmes, whereby effects tend to decline at 1-year follow-up [[4](#_ENREF_4), [9](#_ENREF_9)]. There is a lack of published evidence on the sustainability of effects beyond this period [[9](#_ENREF_9)]. Studies often do not include a control group beyond the first year of follow-up because parents are subsequently offered the intervention.

Effects sizes modelled do not account for imperfect adherence since the studies used to source the effectiveness estimates did not use an ITT approach and only analysed intervention completers. Thus, we assumed that study completers would receive a full effect, while dropouts would incur a cost but receive no benefit. In clinical work with children, 40%–60% drop out of treatment prematurely and hence may not be receiving the benefits of treatment [[20](#_ENREF_20)].

# 3. Identification of the eligible population

The eligible population for the treatment of CD included all children aged 5-9 years in the Australian population. We limited the scope of the model to children currently seeking treatment because the intervention does not include a new case finding component, but rather targets parents of children with CD who are treatment seeking. The selected age group reflects the ages comprised in the trials used to provide the effectiveness estimates. Although the trial on individual Triple P targeted children younger than 5 years, our model applied a lower age limit of 5 years as this reflects the age at which diagnosis of CD can be given in clinical practice. We also accounted for the likelihood of attrition (dropouts) as reported in published studies. One parent per child was assumed in all calculations throughout the model. The steps taken to identify the eligible population are as follows:

**Step 1: 2013 Australian population**

We started with the 2013 Australian population aged 5-9 years (N = 1,455,520) sourced from the Australian Bureau of Statistics [[21](#_ENREF_21)].

**Step 2: Prevalence of Conduct disorder**

Data on the prevalence of Conduct disorder was obtained from the Global Burden of Disease study [[22](#_ENREF_22)]. Data was partitioned by age and sex.

**Step 3: Proportion offered the intervention**

The proportion of children/parents offered the intervention was assumed to be 60% by the Technical Advisory Panel on conduct disorder, composed of clinical experts and researchers in the field. This was thought to be a fairly realistic estimate since the intervention does not include a case finding component, but rather targets parents of children with CD who are treatment seeking. This was to reflect current practice since only approximately 60% of the children with CD in Australia currently assess treatment [[23](#_ENREF_23)].

**Step 4: Proportion agreeing to take up intervention**

An estimate of 60% was used to represent the proportion of parents (1 parent per 1 child) agreeing to take up the intervention (parents that would seek help for their children and initiate the intervention). This estimate was sourced from the most recent Australian Child and Adolescent Survey of Mental Health and Wellbeing [[23](#_ENREF_23)].

**Step 5: Proportion dropping out early**

Estimated as 1 - proportion of completers (42%, 95% CI 36% - 49%) and varied according to the parameter's LCI and HCI. Dropping out early corresponded to the completion of 20% of the intervention - i.e. 2 sessions. According to the papers by Kazdin 1994 [[24](#_ENREF_24)], 1996 [[25](#_ENREF_25)], 1998 [[20](#_ENREF_20)], dropping out early was defined as completing six or fewer treatment sessions of a treatment lasting between 24 - 32 weeks (average 28 weeks), thus approximately 20% of the treatment duration.

**Step 6: Proportion completing intervention**

We conducted a meta-analysis of the outcomes of the three abovementioned studies [[20](#_ENREF_20), [24](#_ENREF_24), [25](#_ENREF_25)] to derive a pooled estimate for the proportion of treatment completers, to be used in the model (see figure 1). Based on this we found that 58% (95% CI 52% - 65%) completed the intervention**.**

**Figure 1.** Forest plot of studies used to calculate the proportion of intervention completers used in the model

**Final eligible population estimate**

We estimated a final N = 4,151 parents of children aged 5-9 years old to complete the intervention, approximately 35% of those parents/children who are treatment seeking offered the intervention, and approximately 21% of the children with CD in the 2013 Australian population. Figure 2 depicts the flow chart of steps taken to select the final eligible population, and table 3 shows these steps discriminated by age and sex.

Figure 2. Flow chart of steps taken to select the final eligible population


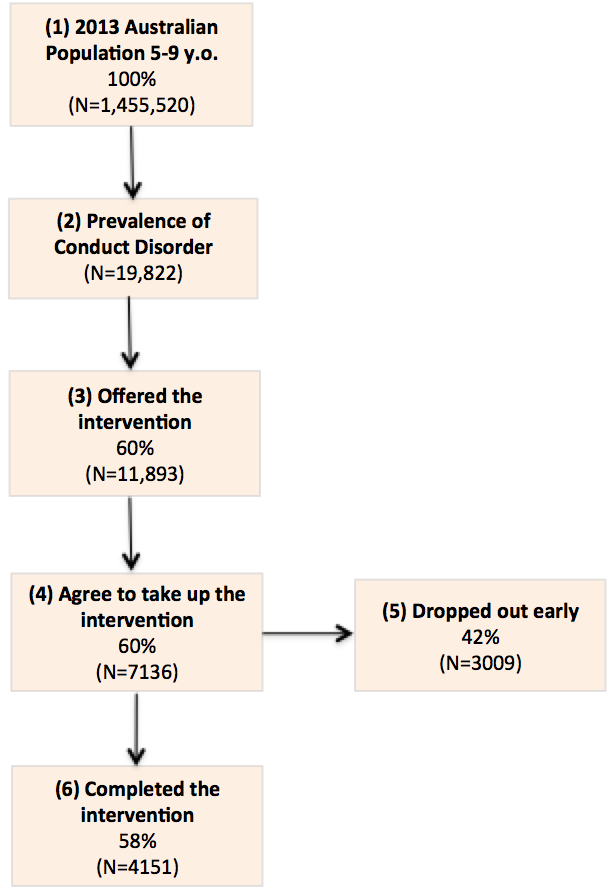


Table 2. Steps taken to select the eligible population discriminated by age and sex

|  | **Step 1: 2013 Australian population** | | **Step 2: Prevalence of Conduct disorder (proportion)** | | **Step 3: Offered the intervention** | | **Step 4: Agree to take up intervention** | | **Step 5: Dropped out early** | | **Step 6: Completed intervention** | |
| --- | --- | --- | --- | --- | --- | --- | --- | --- | --- | --- | --- | --- |
| **Age** | **Male** | **Female** | **Male** | **Female** | **Male** | **Female** | **Male** | **Female** | **Male** | **Female** | **Male** | **Female** |
| **5** | 153,551 | 145,914 | 1,357 | 437 | 814 | 262 | 489 | 157 | 206 | 66 | 284 | 91 |
| **6** | 151,636 | 143,390 | 1,924 | 627 | 1,155 | 376 | 693 | 226 | 292 | 95 | 403 | 131 |
| **7** | 151,356 | 143,442 | 2,786 | 939 | 1,671 | 564 | 1,003 | 338 | 423 | 143 | 583 | 197 |
| **8** | 147,003 | 138,616 | 3,783 | 1,309 | 2,270 | 786 | 1,362 | 471 | 574 | 199 | 792 | 274 |
| **9** | 144,228 | 136,384 | 4,910 | 1,749 | 2,946 | 1,049 | 1,768 | 630 | 745 | 266 | 1,028 | 366 |
| **Sub-total** | 747,774 | 707,776 | 14,760 | 5,061 | 8,856 | 3,037 | 5,314 | 1,822 | 2,241 | 768 | 3,091 | 1,060 |
| **Total** | 1,455,520 | | 19,822 | | 11,893 | | 7,136 | | 3,009 | | 4,151 | |

# 4. Modelling health benefits

## 4.1. Model structure

A population-based multiple cohort Markov model with 1-year cycles was implemented in Excel to estimate the health outcomes resulting from the delivery of either the group or the individual format of the Triple P parenting programme for the treatment of CD among children aged 5-9 years who are treatment seeking. A more complex microsimulation model structure was not deemed relevant, as there was no evidence of variation of effect between discrete individuals. Model cohorts were discriminated by sex and single-year age groups (see Figure 3). Each age-sex cohort was modelled through to 18 years (over a 13 year time horizon), capturing the time period between the age children were given the intervention until adulthood. In this study, the potential continuation of CD into adulthood was not considered, nor were comorbidities or longer-term consequences related to CD, as there is no literature supporting the longer-term effectiveness of Triple P or other parenting programmes in reducing CD or related co-morbidities. CD was modelled along the lines of a chronic, rather than an episodic disorder, as children can only either have the disorder or be free from it. Children were attributed a single disability weight (as per the GBD studies [[5](#_ENREF_5), [26](#_ENREF_26)]) based on the presence or absence of the disorder.

**An adapted Dismod-II model was used to simulate how a population cohort moves between three health states over time - i.e. healthy, diseased and dead [**[**27**](#_ENREF_27)**]. DisMod-II is a computer model with an interactive graphical interface, which is based on a set of differential equations that describe age-specific incidence, remission, case fatality, and 'all other causes' mortality.** In the current treatment scenario, a cohort of children considered healthy (without a diagnosis of CD) was subject to an incidence rate, and could become diseased (prevalent cases of CD in the population). When diseased, the children could be subject to a rate of recovery from the disease - the remission - or to a rate of dying from the disease - the case fatality. Both healthy and diseased children were subject to the same mortality rate from all other causes. The diseased health state included all children with CD; with the prevalence of CD in the initial cycle (i.e., at year 0) being based on the current prevalence of CD in the 2013 Australian population for each respective age-sex cohort, and subsequently impacted by the aforementioned epidemiological parameters (See Figure 4 for state transition diagram). The model calculated annual transitions between health states for each single year of age-sex cohorts staring at age 5 until age 9, until children reached adulthood (i.e., 18 years).

Figure 3. State transition diagram depicting the epidemiological transitions between the three health states considered in the model


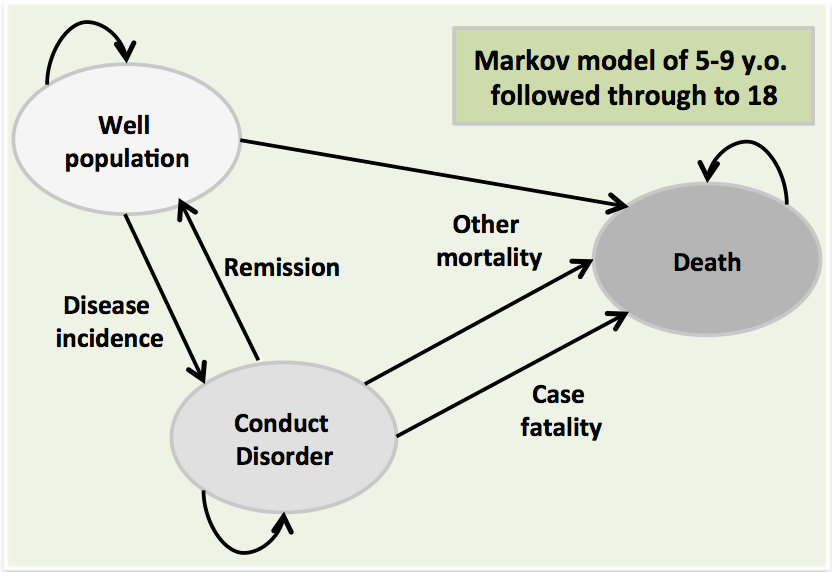


The model assumed that the interventions were fully implemented and operating under “steady state” conditions - i.e. trained staff and necessary infrastructure were available to deliver the intervention, which operated in accordance with its effectiveness potential [[28](#_ENREF_28)].

The model simulates the disease dynamics with and without the delivery of either group or individual Triple P, and estimates the comparative health benefits (i.e., DALYs averted) prevenient from the changes in the disease dynamics of each age-sex cohort over time between two scenarios: (1) the intervention scenario, where the selected eligible population completes the intervention; (2) the “partial null” scenario (the comparator), where the same eligible population receives neither the intervention, Triple P, nor any other treatment currently available within the Australian mental health services. A “partial null” comparator was chosen to represent the theoretical level of disease that could be present if no intervention for this disease were in place, thus equivalent to a “do-nothing” scenario [[29](#_ENREF_29)].

The model calculated the number of years lived with disability in both the intervention and comparator scenarios, whereby the impact of the intervention modelled resulted in the reduction of the number of prevalent cases of CD in the eligible population, i.e. we multiplied the proportion of completers by the intervention effect size (relative risk ratio) at applicable time points over a 13-year time horizon. The model estimated the impact of the intervention on the prevalence of CD in the Australian population, thus the impact on morbidity reflected by the total number of years lived with disability. Only the morbidity component of the DALY was modelled in the current study, as there was no evidence of a mortality impact of CD. Years lived by the cohorts were corrected for morbidity, using CD prevalence and the disability weight from the 2013 GBD study [[22](#_ENREF_22)] The model estimated incremental DALYs averted, reported as the net cost per DALY averted, corresponding to the comparative difference in the total number of DALYs in the intervention and comparator scenarios^[[1]](#footnote-1)^.

## 4.2. Model parameters

### 4.2.1 Incidence

Incidence rates for both males and females aged 5-18 years were obtained for the Australian population from the 2013 GBD study [[22](#_ENREF_22)]. Data are shown in Table 3.

Table 3. Incidence rates for the 2013 Australian population by age and sex

| **Age** | **General Australian population** | |
| --- | --- | --- |
|  | **Males** | **Females** |
| 5 | 0.00479 | \| 0.00189 \| \| --- \| |
| 6 | 0.00788 | 0.00325 |
| 7 | 0.01121 | 0.00478 |
| 8 | 0.01438 | 0.00633 |
| 9 | 0.01711 | 0.00773 |
| 10 | 0.01910 | 0.00883 |
| 11 | 0.02007 | 0.00944 |
| 12 | 0.01974 | 0.00940 |
| 13 | 0.01795 | 0.00862 |
| 14 | 0.01506 | 0.00727 |
| 15 | 0.01153 | 0.00558 |
| 16 | 0.00786 | 0.00381 |
| 17 | 0.00451 | 0.00218 |
| 18 | 0.00189 | 0.00091 |

### 4.2.2 Remission

Remission rates for both males and females aged 5-18 years were obtained for the Australian population from the 2013 GBD study [[22](#_ENREF_22)]. Data are shown in Table 4.

Table 4. Remission rates for the 2013 Australian population by age and sex

| **Age** | **General Australian population** | |
| --- | --- | --- |
|  | **Males** | **Females** |
| 5 | 0.23787 | \| 0.33840 \| \| --- \| |
| 6 | 0.23606 | 0.33307 |
| 7 | 0.23404 | 0.32708 |
| 8 | 0.23209 | 0.32110 |
| 9 | 0.23089 | 0.31626 |
| 10 | 0.23123 | 0.31378 |
| 11 | 0.23389 | 0.31488 |
| 12 | 0.23966 | 0.32080 |
| 13 | 0.24905 | 0.33244 |
| 14 | 0.26158 | 0.34947 |
| 15 | 0.27649 | 0.37122 |
| 16 | 0.29303 | 0.39704 |
| 17 | 0.31046 | 0.42627 |
| 18 | 0.32790 | 0.45783 |

### 4.2.3 Case fatality

Case fatality was considered zero as per the 2013 GBD study [[22](#_ENREF_22)] (this is because no estimate of excess mortality due to CD was found in the literature.

### 4.2.4 All-cause mortality

All-cause mortality rates for both males and females aged 5-18 years were obtained from 2013 population and mortality data sourced from the Australian Bureau of Statistics [[30](#_ENREF_30)]. Data are shown in Table 5.

Table 5. All-cause mortality rates for the 2013 Australian population by age and sex

| **Age** | **General Australian population** | |
| --- | --- | --- |
|  | **Males** | **Females** |
| 5 | 0.00016 | \| 0.00006 \| \| --- \| |
| 6 | 0.00013 | 0.00013 |
| 7 | 0.00007 | 0.00009 |
| 8 | 0.00009 | 0.00006 |
| 9 | 0.00007 | 0.00008 |
| 10 | 0.00007 | 0.00004 |
| 11 | 0.00008 | 0.00009 |
| 12 | 0.00006 | 0.00004 |
| 13 | 0.00017 | 0.00010 |
| 14 | 0.00019 | 0.00013 |
| 15 | 0.00023 | 0.00024 |
| 16 | 0.00024 | 0.00017 |
| 17 | 0.00037 | 0.00023 |
| 18 | 0.00056 | 0.00019 |

### 4.2.5 Prevalence

Prevalence rates for both males and females aged 5-18 years were obtained for the Australian population from the 2013 GBD study [[22](#_ENREF_22)]. Data are shown in Table 6.

Table 6. Prevalence rates for the 2013 Australian population by age and sex

| **Age** | **General Australian population** | |
| --- | --- | --- |
|  | **Males** | **Females** |
| 5 | 0.00737 | \| 0.00250 \| \| --- \| |
| 6 | 0.01303 | 0.00454 |
| 7 | 0.01952 | 0.00698 |
| 8 | 0.02632 | 0.00966 |
| 9 | 0.03305 | 0.01240 |
| 10 | 0.03939 | 0.01503 |
| 11 | 0.04498 | 0.01735 |
| 12 | 0.04948 | 0.01917 |
| 13 | 0.05257 | 0.02034 |
| 14 | 0.05403 | 0.02077 |
| 15 | 0.05364 | 0.02042 |
| 16 | 0.05120 | 0.01922 |
| 17 | 0.04649 | 0.01711 |
| 18 | 0.03954 | 0.01413 |

### 4.2.6 Disability weight

The disability weight was sourced from the 2013 GBD study [[22](#_ENREF_22)]. We used unadjusted disability weight used as the cohort of children modelled in this study are symptomatic and determined appropriate for intervention, therefore the “weighted” disability weight reported in Vos *et al* (2015) is an underestimate, given it includes children with a less severe condition. CD was attributed one single weight. We used a disability weight of 0.241 (95% CI: 0.159 – 0.341).

# 5. Cost analysis

## 5.1. Cost analysis of group and individual Triple P

We developed an intervention pathway that was representative of routine health services delivered within the Australian mental health care system. The intervention pathway for the delivery of face-to-face group Triple P included two main steps: (1) psychological assessment by a general practitioner (first and follow-up visit); and (2) practitioners delivering the intervention (group or individual Triple P) to the eligible participants.

***Psychological assessment by a general practitioner***

Within the Australian mental health care system, children with conduct problems are first referred to a general practitioner (GP) with or without mental health skills training who performs an assessment of the child and makes a referral to a psychologist (first visit). We then assumed that parents would be offered either group or individual Triple P. Upon completion of the course of treatment the child is called for at least one follow-up visit with the GP to review the mental health treatment plan and make necessary adjustments, monitor progress and measure outcomes. We assumed each GP visit to last 1 hour. We costed GP visits using relevant Medicare Benefits Schedule (MBS) fees for each consult type (with or without mental health skills training). We calculated the weighted average unit cost for a consult with an MBS funded GP by weighting individual MBS item fees for each consult type by the relative number of consults in Australia during the year of 2013. Data on the total number of consults for each MBS item during 2013 were obtained from Medicare Australia Statistics [[31](#_ENREF_31)]. Table 7 presents the item numbers and the fees for the first and follow-up visits to a GP.

Table 7. Costs of the first and follow-up visit to a GP

| **Cost of GP Mental Health Treatment - First visit** | | | |
| --- | --- | --- | --- |
| MBS item no. | Service | No. of consults in 2013 | Fee |
| 2700 | First visit (no mental health skills training) | 148,111 | $70.30 |
| 2701 | First visit (no mental health skills training) | 70,920 | $103.50 |
| 2715 | First visit (with mental health skills training) | 476,239 | $89.25 |
| 2717 | First visit (with mental health skills training) | 215,819 | $131.45 |
|  |  |  |  |
| Weighted average | | | $97.27 |

| **Cost of GP Mental Health Treatment - Follow-up visit** | | | |
| --- | --- | --- | --- |
| MBS item no. | Service | No. of consults in 2013 | Fee |
| 2712 | Follow-up visit (reviewing a mental health treatment plan) | 329,040 | $70.30 |

Based on the estimation of the eligible population (see section 3), we estimated that 11,893 parents attended a first GP visit, costing a weighted average of $97.27, and that 4,151 parents attended a follow-up visit, costing $70.30. The total cost of attending a GP was estimated by the sum of the product of the number of parents offered the intervention by the weighted average cost of a first GP visit and the product of the number of parents completing the intervention by the cost of a follow-up GP visit (see Table 8).

***Practitioners delivering the intervention***

In the model, we assumed the interventions, which were the most advanced forms of Triple P, were delivered by psychologists. This was to reflect the common practice within Australian mental health services, as well as the main professional category likely to deliver this level of Triple P.

*Group Triple P*

The delivery of group Triple P included four weekly 2-hour group sessions delivered by two practitioners followed by four weekly individual telephone consultations with an average duration of 30 minutes. Parents were also given a workbook containing the key learning principles of the programme and exercises.

*Individual Triple P*

The delivery of individual Triple P included 10 individual sessions lasting between 60-90 minutes, delivered by one practitioner.

We costed psychologists using relevant MBS fees for each consult type (individual and group-based consults). We made the following assumptions: (1) the cost of a psychologist running group therapy corresponded to a consultation lasting at least 60 minutes; (2) one telephone consultation with a psychologist lasting in average 30 minutes, according to the literature, corresponded to the full cost per consult (lasting at least 50 minutes); (3) the cost of a psychologist running individual therapy (lasting at least 50 minutes) corresponded to one consultation lasting 60 minutes.

We calculated the weighted average unit cost for a consult with an MBS funded psychologist by weighting individual MBS item fees for each consult type by the relative number of consults in Australia during the year of 2013. Data on the total number of consults for each MBS item during 2013 were obtained from Medicare Australia Statistics [[31](#_ENREF_31)]. The cost of the workbooks handed out to parents was sourced from the official Triple P webpage [[32](#_ENREF_32)]. Table 8 presents the item numbers and the fees for individual and group-based consults.

Table 8. Costs of group-and single-based therapy

| **Cost of group therapy service** | | | |
| --- | --- | --- | --- |
| MBS item no. | Service | No. of consults in 2013 | Fee |
| 80020 | Psychological therapy | 11,995 | $37.20 |
| 80120 | Focussed psychological strategies | 18,093 | $25.45 |
|  |  |  |  |
| Weighted average | | | $30.13 |

| **Cost of single psychologist** | | | |
| --- | --- | --- | --- |
| MBS item no. | Service | No. of consults in 2013 | Fee |
| 80010 | Psychological therapy | 1,561,368 | $146.45 |
| 80110 | Focussed psychological strategies | 1,969,233 | $99.75 |
|  |  |  |  |
| Weighted average | | | $120.40 |

Table 9 shows the cost items for group and individual Triple P. We estimated intervention costs for completers and dropouts separately (dropping out early corresponded to the completion of 20% of the intervention - i.e. 2 sessions) for group and individual face-to-face Triple P.

***Intervention costs***

*Group Triple P*

*Completers*

Based on the estimation of the eligible population (see section 3), we estimated that 4,151 parents attended 4 sessions (8h) of group-based Triple P with 2 psychologists, each costing a weighted average of $30.13; 4,151 parents received 4 telephone consultations (2h) with a single psychologist costing a weighted average of $120.40; and 4,151 parents received 2 workbooks costing $35.00 and $14.95. The total cost of attending group Triple P for completers was estimated by adding the total cost of each cost item: cost of group psychologist, cost of single psychologist conducting the telephone consultations, and cost of workbooks. The cost of group-based sessions with 2 psychologists was estimated by the sum of the product of the number of parents completing the intervention by the weighted average cost of a group-based session (times 2 corresponding to 2 psychologists delivering the group-based sessions) and by the total number of sessions offered. The total cost of single psychologist conducting the telephone consultations was estimated by calculating the sum of the product of the number of parents completing the intervention by the weighted average cost of a single psychologist and by the total number of telephone consultations offered. The total cost of the workbooks was estimated by calculating the number of parents completing the intervention by the cost of the workbooks.

Dropouts

We estimated that 3,009 parents attended 4 sessions (8h) of group-based Triple P with 2 psychologists, received 0 telephone consultations and received 2 workbooks. The total cost of attending group Triple P for dropouts was estimated by adding the total cost of each cost item: cost of group psychologist, and cost of workbooks.

*Individual Triple P*

*Completers*

Based on the estimation of the eligible population (see section 3), we estimated that 4,151 parents attended 10 sessions (10h) of individual Triple P with 1 psychologist costing a weighted average of $120.40. The total cost of attending individual Triple P for completers was estimated by the sum of the product of the number of parents completing the intervention by the weighted average cost of single psychologist session and by the total number of sessions offered.

Dropouts

We estimated that 3,009 parents attended 2 sessions (2h) of individual Triple P with 1 psychologist. The total cost of attending individual Triple P for dropouts was estimated by the sum of the product of the number of parents dropping out from the intervention by the weighted average cost of single psychologist session and by the total number of sessions offered.

Table 9. Cost items for group and individual face-to-face Triple P

| **Cost item** | **Unit cost** | **Quantity (n parents)** | **Time (hours)** | **Total** |
| --- | --- | --- | --- | --- |
| **A. Referral by GP** |  |  |  |  |
| GP (First visit) | $97.27 | 11,893 | 1 | $1,156,891 |
| GP (Follow-up visit) | $70.30 | 4,151 | 1 | $291,815 |
|  |  |  |  |  |
| **B. Intervention delivery** |  |  |  |  |
| **Group Triple P (2 facilitators)** |  |  |  |  |
| ***Completers*** |  |  |  |  |
| Psychologist - single (phone calls) | $120.40 | 4,151 | 2 | $999,582 |
| Psychologist - group | $30.13 | 4,151 | 8 | $2,001,400 |
| Workbooks |  |  |  |  |
| *Every parent* | $35.00 | 4,151 | N/A | $145,285 |
| *Every parent group workbook* | $14,95 | 4,151 | N/A | $62,057 |
|  |  |  |  |  |
| ***Dropouts (completing 20% of the intervention)*** |  |  |  |  |
| Psychologist - single (phone calls) | $120.40 | 3,009 | 0 | $0 |
| Psychologist - group | $30.13 | 3,009 | 4 | $725,393 |
| Workbooks |  |  |  |  |
| *Every parent* | $35.00 | 3,009 | N/A | $105,315 |
| *Every parent group workbook* | $14.95 | 3,009 | N/A | $44,985 |
|  |  |  |  |  |
| **Individual Triple P (1 facilitator)** |  |  |  |  |
| ***Completers*** |  |  |  |  |
| Psychologist - single | $120.40 | 4,151 | 10 | $4,997,909 |
|  |  |  |  |  |
| ***Dropouts (completing 20% of the intervention)*** |  |  |  |  |
| Psychologist - single | $120.40 | 3,009 | 2 | $724,582 |

## 5.2. Cost offsets

The baseline analysis included cost offsets, i.e. treatment costs that are avoided due to the reduction in the prevalence of Conduct disorder, accruing to the health sector were included in the base-case analysis. Broader perspectives deemed relevant were included in the sensitivity analysis, as large costs associated with CD fall outside the health care sector. Cost-offsets were estimated from published international literature, as there are currently no Australian estimates. These were divided into health care costs and other sector costs, and estimated for two age cohorts of 5-10 year olds and 11-18 year olds. Other sector costs for the younger cohort included special education, social services and voluntary and private sector costs, while other sector costs for the older cohort included education, foster and residential care, relationships (domestic violence and divorce), state benefits (unemployment and single parenthood) and crime. For the older cohort, cost offsets were sex-adjusted due to data availability. Estimates for the younger cohort of 5-10 year olds were based on data from Edwards et al, 2007 [[33](#_ENREF_33)], Harrington et al, 2000 [[34](#_ENREF_34)] Romeo et al, 2006 [[35](#_ENREF_35)], while estimates for the older cohort of 11-18 year olds were based on data from Scott et al, 2001 [[36](#_ENREF_36)]. All costs were converted into Australian dollars using purchasing power parities (<http://eppi.ioe.ac.uk/costconversion/default.aspx>) and then inflated to 2012-2013 values using the Australian health price deflators [[37](#_ENREF_37)]. This two-step process was required since Australian health price deflators only span back to ten years back from 2012-13. Cost-offsets were calculated by subtracting the costs of CD for each prevalent case averted by the intervention relative to the comparator. Table 10 shows the average annual cost offset for a treated case of conduct disorder by age and sex, where applicable.

Table 10. Average annual cost offset for a treated case of conduct disorder by age and sex

| **Age** | **Health care costs*** | | **Health care and other sector costs*** | |
| --- | --- | --- | --- | --- |
|  | **Males** | **Females** | **Males** | **Females** |
| **5-10** | $1,076.03 | $1,076.03 | $3,346.88 | $3,346.88 |
| **11-18** | $333.01 | $141.03 | $1,168.72 | $11,187.30 |

*All cost estimates: (range: ±20%)

# 6. Sensitivity analyses

To investigate the impact of specific input parameters and assumptions on the model outcomes, univariate sensitivity analyses were performed. We modelled the individual impact of: (1) excluding cost-offsets related to the health care sector; (2) including cost offsets related to both the health care sector and other non-health sectors; (3) including time and travel costs; (4a) assuming a decay rate of 50% in effects after year 1 over 5 years (by year 5 the RR is close to the null); (4b) assuming intervention effects persist over 5 years; (5) assuming dropouts get half of the benefit (50% of the effect size); and (6a and b) applying a 0% and 6% discount rate to both costs and benefits. A more detailed description of each analysis is presented below.

## 6.1. Sensitivity analysis excluding cost offsets related to the health care sector

In this analysis we excluded cost offsets pertaining to the health sector from the calculation of net costs, which led to slightly higher ICERs for both group and individual Triple P compared with the base-case analysis.

## 6.2. Sensitivity analysis including cost offsets related to both the health care sector and other non-health sectors

In this analysis we included cost offsets pertaining to both the health care sector and other non-health sectors (see section 5.2 for a description of the cost included in other sectors). Group Triple P became cost-saving when including cost-offsets pertaining to all sectors (cost-offsets: $10.5M group; $1M individual), whereas the ICER for individual Triple P decreased marginally.

## 6.3. Sensitivity analysis including time and travel costs

Time and travel costs accruing to parents were excluded from the base-case analysis but included in a sensitivity analysis. In the context of the current study, time and travel costs would include the costs of parents to travel to and from: (1) GP assessment visits (first visit and follow-up visit); and (2) group-based and individual-based sessions of Triple P. Time and travel costs were estimated separately for completers and dropouts, and then added together to get total cost estimates. We made the following assumptions: (1) parents who attended a first visit with the GP corresponded with parents who were offered the intervention - i.e. 11,893 parents (Table 2, section 3); (2) parents who attended a follow-up visit with the GP corresponded with parents who completed the interventions - i.e. 4,151 (Table 2, section 3). Completers attended a first visit and a follow-up visit with the GP and the full course of the interventions. Dropouts attended a first visit with the GP and two sessions of the intervention.

We used time and travel unit costs from a previous study by Vos et al ^[^[^28^](#_ENREF_28)^]^. The average time cost per hour was AU$17.44, and the average travel cost per trip was AU$9.96, expressed in 2003 Australian dollars. We used Australian health price deflators from the Australian Institute of Health and Welfare to convert these costs into 2013 Australian dollars ^[^[^38^](#_ENREF_38)^]^. Hence, the average time cost per hour was AU$24.67, and the average travel cost per trip was AU$9.96.

***Travel costs***

To estimate travel costs we assumed: (1) two trips for each visit to the GP (first visit for initial assessment and follow-up visit post-intervention); and (2) two trips for each session to attend either group or individual face-to-face Triple P.

*Group Triple P*

To calculate travel costs for parents *completing* group Triple P we multiplied the average travel cost per trip (AU$9.96) by 2 trips by the sum of all visits/consultations - i.e., number of parents attending a first GP visit (11,893), number of parents attending a follow-up GP visit (4,151), and number of parents attending 4 sessions of group Triple P (4,151 x 4 = 16,604), totalling AU$650,322.08. To calculate travel costs for parents *dropping out* from the intervention, we multiplied the average travel cost per trip (AU$9.96) by 2 trips by the sum of all visits/consultations - i.e., number of parents attending a first GP visit (11,893), and number of parents attending 2 sessions of group Triple P (3,009 x 2 = 8,302), totalling AU$119,877.22. Total travel costs for group Triple P were AU$770,199.30.

*Individual Triple P*

To calculate travel costs for parents *completing* individual Triple P we multiplied the average travel cost per trip (AU$9.96) by 2 trips by the sum of all visits/consultations - i.e., number of parents attending a first GP visit (11,893), number of parents attending a follow-up GP visit (4,151), and number of parents attending 10 sessions of individual Triple P (4,151 x 10 = 41,510), totalling AU$1,146,414.77. To calculate travel costs for parents *dropping out* from the intervention, we multiplied the average travel cost per trip (AU$9.96) by 2 trips by the sum of all consultations - i.e., number of parents attending a first GP visit (11,893), and number of parents attending 2 sessions of individual Triple P (3,009 x 2 = 8,302), totalling AU$119,877.22. Total travel costs for individual Triple P were AU$1,266,291.99.

***Time costs***

To estimate time costs we calculated the product of each consultation by the estimated time per consultation.

*Group Triple P*

To calculate time costs for parents *completing* group Triple P, we multiplied the average time cost per hour (AU$24.67) by the sum of all consultations - i.e. time at the first GP visit (1h x 11,893), time at the follow-up visit with the GP (1h x 4,151), time attending group-based sessions (4 x 2h x 4,151), and time at telephone consultations (4 x 0.5h x 4,151). Total time costs for completers were AU$1,419,781.74. To calculate travel costs for parents *dropping out,* we multiplied the average time cost per hour (AU$24.67) by the sum of all consultations - i.e. time at the first GP visit (1h x 11,893), and time attending group-based sessions (2 x 2h x 3,009). Total time costs for dropouts were AU$296,924,80. Total time costs for group Triple P totalled AU$1,716,706.55.

*Individual Triple P*

To calculate time costs for parents *completing* individual Triple P, we multiplied the average time cost per hour (AU$24.67) by the sum of all consultations - i.e. time at the first GP visit (1h x 11,893), time at the follow-up visit with the GP (1h x 4,151), and time attending individual-based sessions (10 x 1h x 4,151). Total time costs for completers were AU$1,419,781.74. To calculate travel costs for parents *dropping out,* we multiplied the average time cost per hour (AU$24.67) by the sum of all consultations - i.e. time at the first GP visit (1h x 11,893), and time attending individual-based sessions (2 x 1h x 3,009). Total time costs for dropouts were AU$148,462,40. Total time costs for individual Triple P totalled AU$1,716,706.55.

The inclusion of time and travel costs in the sensitivity analysis tripled the ICER for group Triple P, whereas it led to individual Triple P having a slightly higher ICER and a lower probability of being cost-effective.

## 6.4. Sensitivity analysis assuming a 50% decay rate in effect sizes after year 1 over 5 years

In the base-case analysis, we assumed the intervention effects at post-test to be maintained at 1-year follow-up, and the effect to be null in the years thereafter. This is consistent with the literature on the effectiveness of Triple P and other parenting programmes, whereby effects tend to decline at 1-year follow-up [[4](#_ENREF_4), [9](#_ENREF_9)]. Studies reporting longer-term effects are often lacking control groups since they are eventually offered the intervention [[4](#_ENREF_4)]. This was consisted with three studies on the effectiveness of Triple P [[15](#_ENREF_15), [39](#_ENREF_39), [40](#_ENREF_40)] that reported outcomes for the intervention group over a longer follow-up period (up to 2 years) but that did not meet the inclusion criteria of our study. We plotted the outcomes of those studies to try to identify a pattern of intervention effect. We identified that both the intervention and the control group improved over time, and the difference in effect between conditions seemed to be maintained over time. To reflect the decay in effect over time observed in the literature we used an annual decay rate of 50% in effect after year 1 (by year 5 the RR is close to the null) that was modelled for both the intervention and the no-intervention scenario in a sensitivity analysis.

Assuming a 50% annual decay rate of the effect size led to group Triple P becoming cost-saving, but with a wide uncertainty interval which intersected the south-east, north-east, and north-west quadrants of the plane, i.e. being either cost-saving (dominant) or economically inefficient (dominated). This scenario also resulted in individual Triple P being more cost-effective, but with a wide uncertainty interval spanning over both the north-west and the north-east quadrants of the plane - i.e. with a double likelihood of being less effective and more costly than the comparator (dominated), and more costly and more effective than the comparator. This great uncertainty is due to the wide confidence interval of the effect size that becomes non-significant when assuming a 50% decay over time. This is reflected on the large variation in the total DALYs.

## 6.5. Sensitivity analysis assuming intervention effects persist over 5 years

We tested further the impact of a different assumption on the effect sizes of group and individual Triple P and assumed the effects persisted over 5 years, being null thereafter. The cost-effectiveness of group and individual therapy improved greatly when assuming intervention effects persisted over 5 years, with group Triple P becoming cost-saving.

## 6.6. Sensitivity analysis assuming dropouts get half of the benefit (50% of the effect size)

In the base-case analysis we made a conservative approach, and assumed dropouts incurred a cost but no benefit. In clinical work with children, 40%–60% drop out of treatment prematurely and hence may not be receiving the benefits of treatment [[20](#_ENREF_20)]. However, in a paper by Kazdin et al, 1998, although treatment completion was strongly related to therapeutic change, many dropouts (34%) improved and improvement was predicted by similar variables whether or not children completed treatment. With this in mind, we decided to evaluate the impact of attributing dropouts half of the benefits attributed to intervention completers in a sensitivity analysis. The cost-effectiveness results for both interventions differed little from the base-case when assuming dropouts get 50% of the health benefits.

## 6.7. Sensitivity analysis applying different discount rates to costs and benefits

In the base-case analysis we used discount rates of 3%, which were applied equally to both costs and health benefits. We tested the impact of applying different discount rates (0% and 6%) to both costs and health benefits on the model results. The cost-effectiveness results for both interventions differed little from the base-case when assuming differential discount rates.

# 7. Consolidated Health Economic Evaluation Reporting Standards (CHEERS) statement

| **Section/item** | **Item No** | **Recommendation** | **Reported on page No/ line No** |
| --- | --- | --- | --- |
| **Title and abstract** | | | |
| Title | 1 | Identify the study as an economic evaluation or use more specific terms such as “cost-effectiveness analysis”, and describe the interventions compared. | Page 1 |
| Abstract | 2 | Provide a structured summary of objectives, perspective, setting, methods (including study design and inputs), results (including base case and uncertainty analyses), and conclusions. | Page 2 |
| **Introduction** | | | |
| Background and objectives | 3 | Provide an explicit statement of the broader context for the study. Present the study question and its relevance for health policy or practice decisions | Page 3, line 25  Page 4, lines 1-12  Appendix section 1 |
| **Methods** | | | |
| Target population and subgroups | 4 | Describe characteristics of the base case population and subgroups analysed, including why they were chosen. | Page 7, lines 2-11 |
| Setting and location | 5 | State relevant aspects of the system(s) in which the decision(s) need(s) to be made. | Page 4, lines 16-17 |
| Study perspective | 6 | Describe the perspective of the study and relate this to the costs being evaluated. | Page 4, lines 18-20  Page 8, lines 12-20 |
| Comparators | 7 | Describe the interventions or strategies being compared and state why they were chosen. | Page 4, lines 22-24  Page 6, lines 2-3 |
| Time horizon | 8 | State the time horizon(s) over which costs and consequences are being evaluated and say why appropriate. | Page 8, lines 11-13  Appendix section 4.1 |
| Discount rate | 9 | Report the choice of discount rate(s) used for costs and outcomes and say why appropriate. | Page 4, lines 16-17  Page 5, lines 2-3 |
| Choice of health outcomes | 10 | Describe what outcomes were used as the measure(s) of benefit in the evaluation and their relevance for the type of analysis performed. | Page 4, lines 16-24  Page 5, line 1 |
| Measurement of effectiveness | 11a | *Single study-based estimates:*Describe fully the design features of the single effectiveness study and why the single study was a sufficient source of clinical effectiveness data. | Not applicable |
|  | 11b | *Synthesis-based estimates*: Describe fully the methods used for identification of included studies and synthesis of clinical effectiveness data. | Page 5, lines 6-22  Page 6, lines 1-24  Page 8, lines 22-23  Appendix section 2 |
| Measurement and valuation of preference based outcomes | 12 | If applicable, describe the population and methods used to elicit preferences for outcomes. | Not applicable |
| Estimating resources and costs | 13a | *Single study-based economic evaluation:* Describe approaches used to estimate resource use associated with the alternative interventions. Describe primary or secondary research methods for valuing each resource item in terms of its unit cost. Describe any adjustments made to approximate to opportunity costs. | Not applicable |
|  | 13b | *Model-based economic evaluation:*Describe approaches and data sources used to estimate resource use associated with model health states. Describe primary or secondary research methods for valuing each resource item in terms of its unit cost. Describe any adjustments made to approximate to opportunity costs. | Page 9, lines 1-22  Appendix section 5 |
| Currency, price date, and conversion | 14 | Report the dates of the estimated resource quantities and unit costs. Describe methods for adjusting estimated unit costs to the year of reported costs if necessary. Describe methods for converting costs into a common currency base and the exchange rate. | Page 4, line 24  Page 5, lines 1-2  Appendix section 5.2 |
| Choice of model | 15 | Describe and give reasons for the specific type of decision-analytical model used. Providing a figure to show model structure is strongly recommended. | Page 8, lines 1-17  Appendix section 4.1 (full description) |
| Assumptions | 16 | Describe all structural or other assumptions underpinning the decision-analytical model. | Appendix sections 2.4, 3, 4.1, 5 |
| Analytical methods | 17 | Describe all analytical methods supporting the evaluation. This could include methods for dealing with skewed, missing, or censored data; extrapolation methods; methods for pooling data; approaches to validate or make adjustments (such as half cycle corrections) to a model; and methods for handling population heterogeneity and uncertainty. | Page 10, lines 1-15 |
| **Results** | | | |
| Study parameters | 18 | Report the values, ranges, references, and, if used, probability distributions for all parameters. Report reasons or sources for distributions used to represent uncertainty where appropriate. Providing a table to show the input values is strongly recommended. | Tables 1-2 in main manuscript |
| Incremental costs and outcomes | 19 | For each intervention, report mean values for the main categories of estimated costs and outcomes of interest, as well as mean differences between the comparator groups. If applicable, report incremental cost-effectiveness ratios. | Table 3 in main manuscript |
| Characterising uncertainty | 20a | *Single study-based economic evaluation:* Describe the effects of sampling uncertainty for the estimated incremental cost and incremental effectiveness parameters, together with the impact of methodological assumptions (such as discount rate, study perspective). | Not applicable |
|  | 20b | *Model-based economic evaluation:*Describe the effects on the results of uncertainty for all input parameters, and uncertainty related to the structure of the model and assumptions. | Table 4 in main manuscript |
| Characterising heterogeneity | 21 | If applicable, report differences in costs, outcomes, or cost-effectiveness that can be explained by variations between subgroups of patients with different baseline characteristics or other observed variability in effects that are not reducible by more information. | Not applicable |
| **Discussion** | | | |
| Study findings, limitations, generalisability, and current knowledge | 22 | Summarise key study findings and describe how they support the conclusions reached. Discuss limitations and the generalisability of the findings and how the findings fit with current knowledge. | Page 11-16 |
| **Other** | | | |
| Source of funding | 23 | Describe how the study was funded and the role of the funder in the identification, design, conduct, and reporting of the analysis. Describe other non-monetary sources of support. | Page 16-17 “Funding” |
| Conflicts of interest | 24 | Describe any potential for conflict of interest of study contributors in accordance with journal policy. In the absence of a journal policy, we recommend authors comply with International Committee of Medical Journal Editors recommendations. | Page 2 “Declaration of interest” |

The CHEERS statement checklist format is based on the format of the CONSORT statement checklist

# 7. References

1. Sanders MR (1999) Triple P-Positive Parenting Program: towards an empirically validated multilevel parenting and family support strategy for the prevention of behavior and emotional problems in children. Clin Child Fam Psychol Rev 2:71-90

2. Sanders MR (2012) Development, evaluation, and multinational dissemination of the triple P-Positive Parenting Program. Annu Rev Clin Psychol 8:345-379

3. National Health and Medical Research Council (2009) NHMRC levels of evidence and grades for recommendations for developers of guidelines. In:

4. Sanders MR, Kirby, J. N., Tellegen, C. L., Day, J. J. (2014) The Triple P-Positive Parenting Program: A systematic review and meta-analysis of a multi-level system of parenting support.337-357

5. Erskine HE, Ferrari AJ, Nelson P, Polanczyk GV, Flaxman AD, Vos T, Whiteford HA, Scott JG (2013) Epidemiological modelling of attention-deficit/hyperactivity disorder and conduct disorder for the Global Burden of Disease Study 2010. J Child Psychol Psychiatry 54:1263-1274

6. Erskine HE, Ferrari AJ, Polanczyk GV, Moffitt TE, Murray CJ, Vos T, Whiteford HA, Scott JG (2014) The global burden of conduct disorder and attention-deficit/hyperactivity disorder in 2010. J Child Psychol Psychiatry 55:328-336

7. Nowak C, Heinrichs N (2008) A comprehensive meta-analysis of Triple P-Positive Parenting Program using hierarchical linear modeling: effectiveness and moderating variables. Clinical Child and Family Psychology Review, 11:114-144

8. de Graaf I, Speetjens P, Smit F, de Wolff M, Tavecchio L (2008) Effectiveness of the Triple P Positive Parenting Program on behavioral problems in children: a meta-analysis. Behav Modif 32:714-735

9. Furlong M, McGilloway S, Bywater T, Hutchings J, Smith SM, Donnelly M (2012) Behavioural and cognitive-behavioural group-based parenting programmes for early-onset conduct problems in children aged 3 to 12 years. Cochrane Database Syst Rev 2:CD008225

10. Frank TJ, Keown LJ, Sanders MR (2015) Enhancing Father Engagement and Interparental Teamwork in an Evidence-Based Parenting Intervention: A Randomized-Controlled Trial of Outcomes and Processes. Behav Ther 46:749-763

11. Reese RJ, Slone NC, Soares N, Sprang R (2015) Using telepsychology to provide a group parenting program: A preliminary evaluation of effectiveness. Psychological Services 12:274-282

12. Markie-Dadds C, Sanders MR (2006a) A controlled evaluation of an enhanced self-directed behavioural family intervention for parents of children with conduct problems in rural and remote areas. Behaviour Change:55-72

13. Markie-Dadds C, Sanders MR (2006b) Self-Directed Triple P (Positive Parenting Program) for mothers with children at-risk of developing conduct problems. . Behavioural and Cognitive Psychotherapy:259-275

14. Martin AJ, Sanders MR (2003) Balancing work and family: A controlled evaluation of the Triple P-Positive Parenting Program as a work-site intervention. . Child and Adolescent Mental Health:161-169

15. Sanders MR, Baker S, Turner KM (2012) A randomized controlled trial evaluating the efficacy of Triple P Online with parents of children with early-onset conduct problems. Behav Res Ther 50:675-684

16. Sanders MR, Markie-Dadds C, Tully LA, Bor W (2000) The triple P-positive parenting program: a comparison of enhanced, standard, and self-directed behavioral family intervention for parents of children with early onset conduct problems. J Consult Clin Psychol 68:624-640

17. Connell S, Sanders MR, Markie-Dadds C (1997) Self-directed behavioral family intervention for parents of oppositional children in rural and remote areas. Behav Modif 21:379-408

18. Eyberg SM, Pincus D (1999) Eyberg Child Behavior Inventory and Sutter-Eyberg Student Behavior Inventory: Professional manual. Psychological Assessment Resources, Odessa, FL

19. Sanders MR, Bor W, Morawska A (2007) Maintenance of treatment gains: a comparison of enhanced, standard, and self-directed Triple P-Positive Parenting Program. J Abnorm Child Psychol 35:983-998

20. Kazdin AE, Wassell G (1998) Treatment completion and therapeutic change among children referred for outpatient therapy. Professional Psychology: Research and Practice 29:332-340

21. ABS (2013) Australian Demographic Statistics. In: Report 3101.0. Australian Bureau of Statistics

22. DALYs GBD, Collaborators H, Murray CJ, Barber RM, Foreman KJ, Abbasoglu Ozgoren A, Abd-Allah F, Abera SF, Aboyans V, Abraham JP, Abubakar I, Abu-Raddad LJ, Abu-Rmeileh NM, Achoki T, Ackerman IN, Ademi Z, Adou AK, Adsuar JC, Afshin A, Agardh EE, Alam SS, Alasfoor D, Albittar MI, Alegretti MA, Alemu ZA, Alfonso-Cristancho R, Alhabib S, Ali R, Alla F, Allebeck P, Almazroa MA, Alsharif U, Alvarez E, Alvis-Guzman N, Amare AT, Ameh EA, Amini H, Ammar W, Anderson HR, Anderson BO, Antonio CA, Anwari P, Arnlov J, Arsic Arsenijevic VS, Artaman A, Asghar RJ, Assadi R, Atkins LS, Avila MA, Awuah B, Bachman VF, Badawi A, Bahit MC, Balakrishnan K, Banerjee A, Barker-Collo SL, Barquera S, Barregard L, Barrero LH, Basu A, Basu S, Basulaiman MO, Beardsley J, Bedi N, Beghi E, Bekele T, Bell ML, Benjet C, Bennett DA, Bensenor IM, Benzian H, Bernabe E, Bertozzi-Villa A, Beyene TJ, Bhala N, Bhalla A, Bhutta ZA, Bienhoff K, Bikbov B, Biryukov S, Blore JD, Blosser CD, Blyth FM, Bohensky MA, Bolliger IW, Bora Basara B, Bornstein NM, Bose D, Boufous S, Bourne RR, Boyers LN, Brainin M, Brayne CE, Brazinova A, Breitborde NJ, Brenner H, Briggs AD, Brooks PM, Brown JC, Brugha TS, et al. (2015) Global, regional, and national disability-adjusted life years (DALYs) for 306 diseases and injuries and healthy life expectancy (HALE) for 188 countries, 1990-2013: quantifying the epidemiological transition. Lancet 386:2145-2191

23. Lawrence D, Johnson S, Hafekost J, Boterhoven de Haan K, Sawyer M, Ainley J, Zubrick SR (2015) The Mental Health of Children and Adolescents. Report on the second Australian Child and Adolescent Survey of Mental Health and Wellbeing. In: Health Do (ed)Commonwealth of Australia, Canberra

24. Kazdin AE, Mazurick JL (1994) Dropping out of child psychotherapy: distinguishing early and late dropouts over the course of treatment. J Consult Clin Psychol 62:1069-1074

25. Kazdin AE (1996) Dropping Out of Child Psychotherapy: Issues for Research and Implications for Practice. Clinical Child Psychology and Psychiatry 1:133-156

26. Global Burden of Disease Study C (2015) Global, regional, and national incidence, prevalence, and years lived with disability for 301 acute and chronic diseases and injuries in 188 countries, 1990-2013: a systematic analysis for the Global Burden of Disease Study 2013. Lancet 386:743-800

27. Barendregt JJ, Van Oortmarssen GJ, Vos T, Murray CJ (2003) A generic model for the assessment of disease epidemiology: the computational basis of DisMod II. Popul Health Metr 1:4

28. Vos T, Carter R, Barendregt JJ, Mihalopoulos C, Veerman JL, Magnus A, Team A-P (2010) Assessing Cost-Effectiveness in Prevention (ACE-Prevention): Final Report. In:University of Queensland, Brisbane and Deakin University, Melbourne

29. Tan-Torres Edejer T, Baltussen R, Adam T, Hutubessy R, Acharya A, Evans DB, Murray CJL (2003) Making choices in health: WHO guide to cost-effectiveness analysis. In:World Health Organization, Geneva

30. ABS (2013) Deaths, Australia, 2013. In: Report 3302.0. Australian Bureau of Statistics

31. Department of Human Services Medicare Item Reports. In:Australian Government

32. Triple P (Positive Parenting Program) In:

33. Edwards RT, Ceilleachair A, Bywater T, Hughes DA, Hutchings J (2007) Parenting programme for parents of children at risk of developing conduct disorder: cost effectiveness analysis. BMJ 334:682

34. Harrington R, Peters S, Green J, Byford S, Woods J, McGowan R (2000) Randomised comparison of the effectiveness and costs of community and hospital based mental health services for children with behavioural disorders. Brittish Medical Journal 321:1047-1050

35. Romeo R, Knapp M, Scott S (2006) Economic cost of severe antisocial behaviour in children--and who pays it. Brittish Journal of Psychiatry 188:547-553

36. Scott S, Knapp, M., Henderson, J., Maughan, B. (2001) Financial cost of social exclusion: follow up study of antisocial children into adulthood. BMJ 323:191-194

37. Australian Institute of Health and Welfare (2014) Health Expenditure Australia 2012-13. In:Australian Institute of Health and Welfare,, Canberra

38. AIHW (2014) Health expenditure Australia 2012-13. In: Welfare AIoHa (ed), Canberra

39. Bodenmann G, Cina A, Ledermann T, Sanders MR (2008) The efficacy of the Triple P-Positive Parenting Program in improving parenting and child behavior: a comparison with two other treatment conditions. Behav Res Ther 46:411-427

40. Zubrick SR, Ward KA, Silburn SR, Lawrence D, Williams AA, Blair E, Robertson D, Sanders MR (2005) Prevention of child behavior problems through universal implementation of a group behavioral family intervention. Prev Sci 6:287-304

1. The concept of DALYs averted, as used in economic evaluation studies, holds a different qualitative interpretation to DALYs, as used in conventional burden of disease studies. In the former, DALYs averted are a measure of health gain with a similar interpretation to quality-adjusted life years (QALYs) – i.e., a greater number of DALYs averted denotes a greater health gain. By contrast, DALYs used in burden of disease studies denote a measure of health loss where a larger number of DALYs denotes a greater disease burden. [↑](#footnote-ref-1)
